# A pro-inflammatory metastasis-associated macrophage subset induces tumor-promoting mesothelial cell conversion in ovarian cancer via IL-1α secretion

**DOI:** 10.1038/s41419-026-09280-1

**Published:** 2026-09-12

**Authors:** Sophie Heidemann, Florian Finkernagel, Anna Mary Steitz, Julie Koennecke, Franziska Werner, Vanessa M. Beutgen, Witold Szymanski, Johannes Graumann, Julia Teply-Szymanski, Andrea Nist, Thorsten Stiewe, Corinna U. Keber, Niklas Gremke, Uwe Wagner, Carsten Denkert, María Gómez-Serrano, Elke Pogge von Strandmann, Rolf Müller, Silke Reinartz

**Affiliations:** 1 Clinic for Gynecology, Gynecological Oncology and Gynecological Endocrinology, Center for Tumor Biology and Immunology, Philipps-Universität Marburg, Marburg, Germany; 2 Genomics Core Facility, Philipps-Universität Marburg, Marburg, Germany; 3 Institute of Translational Proteomics & Core Facility Translational Proteomics, Philipps-Universität Marburg, Marburg, Germany; 4 Institute for Pathology, Philipps-Universität Marburg and University Hospital Marburg (UKGM), Marburg, Germany; 5 Institute of Molecular Oncology, Member of the German Center for Lung Research (DZL), Philipps-Universität Marburg, Marburg, Germany; 6 Clinic for Gynecology, Gynecological Oncology and Gynecological Endocrinology, University Hospital Marburg (UKGM), Marburg, Germany; 7 Institute for Tumor Immunology, Center for Tumor Biology and Immunology, Philipps-Universität Marburg, Marburg, Germany; 8 Present Address: Institute of Pharmaceutical Biology, Goethe University Frankfurt, Frankfurt am Main, Germany; 9 Present Address: Clinical Cooperation Unit Applied Tumor Immunity, German Cancer Research Center (DKFZ), Heidelberg, Germany

**Keywords:** Cancer microenvironment, Immunoediting

## Abstract

Tumor-associated macrophages (TAMs) are key regulators of the tumor microenvironment, yet the functional specialization of TAM subsets in metastatic progression remains incompletely defined. Here, we characterized distinct TAM populations contributing to tumor-promoting mesothelial cell conversion in high-grade ovarian carcinoma using single-cell RNA sequencing of patient-derived macrophages from ascites (ascTAMs) and omental metastases (omTAMs). TAMs from these anatomical sites were clearly distinguishable by polarization states, with omTAMs exhibiting a mixed M1⁺/M2⁺ phenotype, in contrast to the M1^low^/M2⁺ profile observed in ascTAMs. Transcriptomic analysis further revealed functional divergence of these subsets. Notably, omTAMs displayed gene signatures associated with mesothelial-to-mesenchymal transition (MMT), a critical process enabling tumor invasion across the peritoneal lining. Functionally, conditioned media from omTAMs, similar to that from classically activated M1 macrophages, induced MMT in primary mesothelial cells via TGFβ and ERK/p38 MAPK signaling pathways. This phenotypic transition enhanced transmesothelial tumor cell invasion. Proteomic analysis identified IL-1α as a key MMT-inducing factor secreted by pro-inflammatory macrophages. Mechanistically, IL-1α cooperates with TGFβ by activating an autocrine TGFβ/TGFBR1 feedback loop in mesothelial cells, thereby amplifying MMT. Consistent with these findings, IL1A expression was enriched in omTAM clusters across independent patient samples and was confirmed by immunohistochemical analysis of clinical samples. From a therapeutic perspective, our study identifies new avenues to counteract the mesothelial reprogramming driven by IL-1α⁺ TAMs, potentially impeding metastatic progression.

**Figure:**
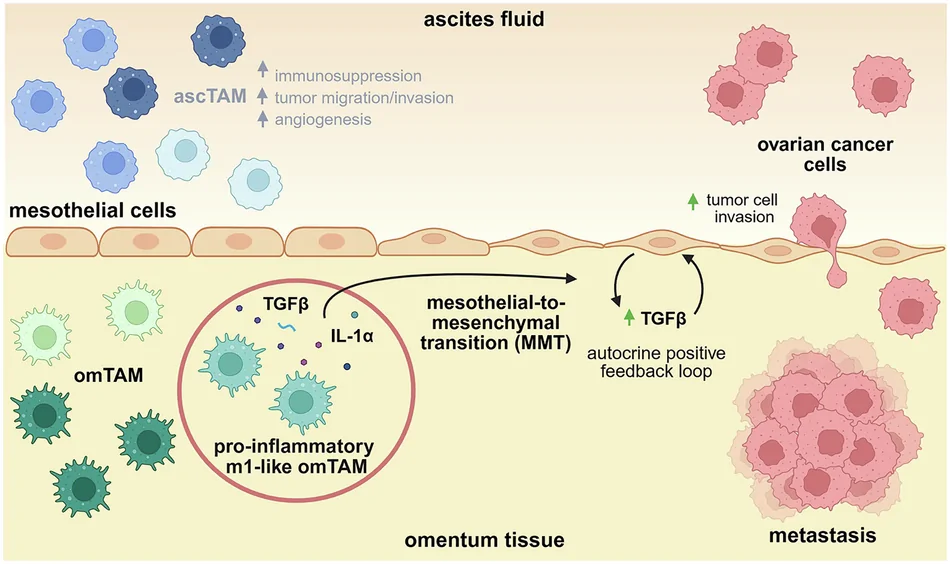
Created in BioRender. Heidemann, S. (2026) https://BioRender.com/aeu6yd0.

Created in BioRender. Heidemann, S. (2026) https://BioRender.com/aeu6yd0.

## Introduction

High-grade ovarian cancer (OC) is an aggressive malignancy typically diagnosed at advanced stages, when malignant ascites has accumulated and widespread peritoneal metastases have formed, preferentially within the lipid-rich omentum. The tumor microenvironment (TME) in OC is characterized by a substantial immune cell infiltrate, particularly tumor-associated macrophages (TAMs), which are abundant in both ascitic fluid and omental metastases. The omentum is of particular relevance, as CD68⁺ TAM density correlates with disease burden [1], and macrophage-rich milky spots facilitate tumor colonization [2]. TAMs display pronounced heterogeneity and plasticity, encompassing diverse subpopulations with pro- and anti-tumor functions extending beyond the classical M1/M2 dichotomy and instead form a continuum of polarization states [3, 4]. Advances in single-cell RNA sequencing (scRNAseq) have substantially refined the characterization of TAM phenotypes within the TME [5–7]. However, functional roles and spatial organization of molecularly defined TAM subsets remain incompletely understood.

Pro-tumorigenic activities, including enhanced proliferation, migration, invasion, extracellular matrix (ECM) remodeling, epithelial-to-mesenchymal transition (EMT) and immunosuppression, have largely been attributed to M2-like TAM subsets [8–10]. In OC, increased M2 marker expression have been associated with early relapse and poor prognosis, whereas a predominance of M1-like TAMs and a high M1/M2 ratio has been linked to improved survival [11–14]. Nevertheless, accumulating evidence indicates that M1-like TAMs exert context-dependent and functionally diverse roles, and cannot be uniformly regarded as anti-tumorigenic [15]. For instance, pro-inflammatory TAM infiltration with an M1-like phenotype has been associated with aggressive clinical features in breast cancer [16]. Consistent with this, our previous bulk RNA analyses demonstrated a shift toward a pro-inflammatory TAM phenotype in omental metastases as compared to ascitic-derived TAMs, suggesting a role for pro-inflammatory polarization during early metastatic progression [17]. Despite these observations, the contribution of pro-inflammatory TAM subsets to tumor progression remains insufficiently defined. Experimental models using M1-polarized THP-1 macrophages have shown that pro-inflammatory signaling may promote tumor invasion via TNFR-NFκB-mediated upregulation of hyaluronan synthase 2 (HAS2) [18], yet validation in clinical specimens remains lacking.

In addition to immune cells, peritoneal mesothelial cells (MESO) are critical components of the OC TME and play a central role in peritoneal dissemination, a hallmark of this disease [19]. Under physiological conditions, MESO form a protective monolayer lining the abdominal cavity and contribute to tissue repair, homeostasis, fluid regulation and immune surveillance. However, this mesothelial barrier is disrupted under pathological conditions such as chronic inflammation, fibrosis, peritoneal dialysis and cancer [20]. In advanced OC, tumor cells disseminate primarily via ascitic fluid and preferentially colonize the omentum. Consequently, breaching the mesothelial layer represents a critical step preceding tumor invasion into peritoneal tissues [19, 21]. Mesothelial barrier dysfunction may result from multiple processes, including cellular senescence [22], apoptosis [23, 24] and mesothelial-to-mesenchymal transition (MMT), a specific form of EMT [25–27]. While tumor-derived TGFβ has been identified as a key driver of MMT in OC [26, 28] and other malignancies such as gastric cancer [29, 30], the contribution of immune cell-derived factors to MMT induction remains largely unexplored.

Given the established roles of pro-inflammatory mediators such as TGFβ, TNFα and IL-1 in driving MMT [31–33], we hypothesized that OC metastases are enriched in M1-like TAM subsets that promote MMT and thereby facilitate early tumor invasion. To address this, we performed a comparative scRNA-seq analysis of TAMs derived from omental metastases and ascites, aiming to delineate the spectrum of TAM polarization states across distinct compartments of the OC TME. Our analysis identified metastasis-associated TAM clusters exhibiting a mixed M1⁺/M2⁺ transcriptional profile with previously uncharacterized functions. The partial overlap of these clusters with MMT-associated gene signatures prompted further investigation into their role in mesothelial reprogramming. Using primary co-culture systems of MESO cells and macrophages, combined with affinity proteomics, we identified macrophage-derived factors and signaling pathways that drive MMT, thereby providing mechanistic insight into the contribution of pro-inflammatory TAM subsets to early metastatic progression in OC.

## Results

### Identification of M1-like TAM clusters with MMT-driver signatures in OC patients by scRNA-seq

**Fig. 1:**
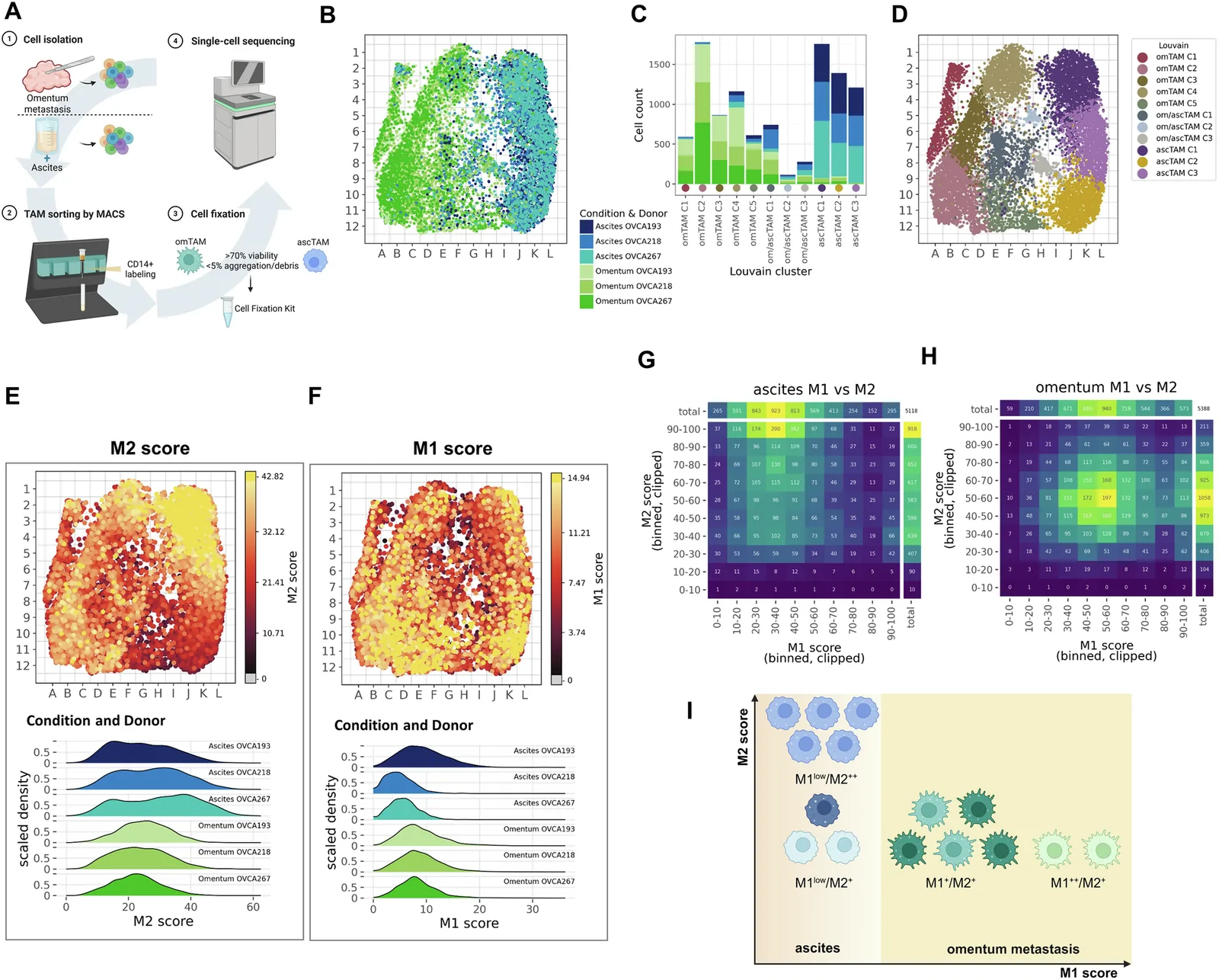
Signatures of TAM clusters from ascites and omental metastases from OC patients. **A** Experimental setup for scRNA-seq of TAM from OC patients: Cells were isolated from matched ascites samples and omental metastatic tissue of three OC-patients, and enriched for CD14+ ascTAM and omTAM by MACS separation. Cells were fixed with Evercode cell fixation kit and frozen for bulk preparation of libraries. Sequencing was performed on an Illumina NovaSeq 550. Created in BioRender. Heidemann, S. (2026) https://BioRender.com/aeu6yd0. **B** Harmony-integrated UMAP projection depicting scRNA-seq data of TAM derived from matched pairs of ascites and omentum of three OC patients (OVCA193, 218, 267) colored by patient and cell source. **C** Distribution of samples stratified by patient, cell source and cell count within Louvain clusters (see Fig. 1D). Cluster names indicate the main cellular source in the cluster (omTAM and ascTAM). Clusters labeled with om/ascTAM comprise omTAM and ascTAM with similar marker gene expression. **D** Louvain clustering of scRNA-seq data of TAM (*n* = 11 clusters). **E, F ***Upper row:* UMAP projections of scRNA-seq data of omTAM and ascTAM from three OC patients colored by M2 score (**E**) and M1 score (**F**). Scores are defined by expression of selected genes associated with M1 and M2 states (Supplementary Table S1). Scores are calculated as sum of (normalized, log2 transformed) genes. The color bar is clipped at the 95th percentile. *Lower row*: Density estimate of M2 and M1 score (as in Fig. 1B) per patients and cell source. **G, H **2D heatmap for quantification of overlapping M1 / M2 score expression in TAM isolated from ascites (**G**) and omentum (**H**). Sum of normalized, log2 transformed expression values are clipped at 95% percentile of all cells and binned by percentile. 2D histograms comprise cell numbers and color scale. The total values are given on the edges. Bin threshholds are constant across panels. **I** Schematic illustration of TAM phenotype distribution in ascites and omentum based on the M1/M2 score expression from Fig. 1G and H. Created in BioRender. Heidemann, S. (2026) https://BioRender.com/aeu6yd0.

To characterize the molecular and functional heterogeneity of TAM subsets across distinct anatomical compartments of the TME, we performed scRNA-seq on matched CD14-enriched ascites-derived TAMs (ascTAMs) and omentum-derived TAMs (omTAMs) from three OC patients, following the workflow outlined in Fig. 1A. A Harmony-integrated UMAP of 10 506 cells, based on highly variable genes and excluding T cell and MESO contaminants, revealed distinct macrophage clusters stratified by cellular origin (ascites and omentum) and patient (OVCA193, 218 and 267) (Fig. 1B). Louvain clustering identified 11 transcriptionally distinct TAM clusters, with five omTAM clusters (omTAM C1-C5) clearly separated from three ascTAM-specific clusters (ascTAM C1-C3), and only minimal overlap at the interface (om/ascTAM C1-3) (Fig. 1C, D). These findings indicate a compartment-specific TAM transcriptional landscape that is consistent across patients.

Analysis of the top five marker genes per cluster suggested functional specialization of TAM subsets (Supplementary Fig. S1 A). ascTAMs predominantly expressed M2-associated markers, including *CD163* and *MARCO* (ascTAM C1), *SIGLEC1* (ascTAM C3), *VCAN* and *CD300E* (ascTAM C2), consistent with immunosuppressive and tissue-remodeling functions. In contrast, omTAM clusters displayed features linked to inflammation and proliferation, including enrichment of chaperone-related genes (omTAM C3), *PLAUR* and *OLR1* (omTAM C5) and cell cycle-associated genes such as *MKI67, CIT and DIAPH3* (omTAM C1), indicative of self-renewal capacity.

To further define polarization states, TAM subsets were scored using established M1 and M2 gene signatures ([34]; Supplementary Table S1). Cells with high M2 scores clustered predominantly in ascTAM C1, whereas lower M2 expression was broadly distributed across omTAM clusters in a patient-independent manner (Fig. 1E; Supplementary Fig. S1 B). In contrast, elevated M1 scores were characteristic of omTAMs, particularly clusters omTAM C2 and C5, while M1-associated expression in ascTAMs was largely restricted to a single patient (OVCA193) (Fig. 1F; Supplementary Fig. S1 C). Co-expression analysis revealed a predominance of M2^high+^/M1^low^ cells in ascites-derived TAMs (Fig. 1G), whereas omTAMs exhibited substantial M1/M2 co-expression (Fig. 1H), indicating a mixed polarization state in metastatic sites (Fig. 1I).

**Fig. 2:**
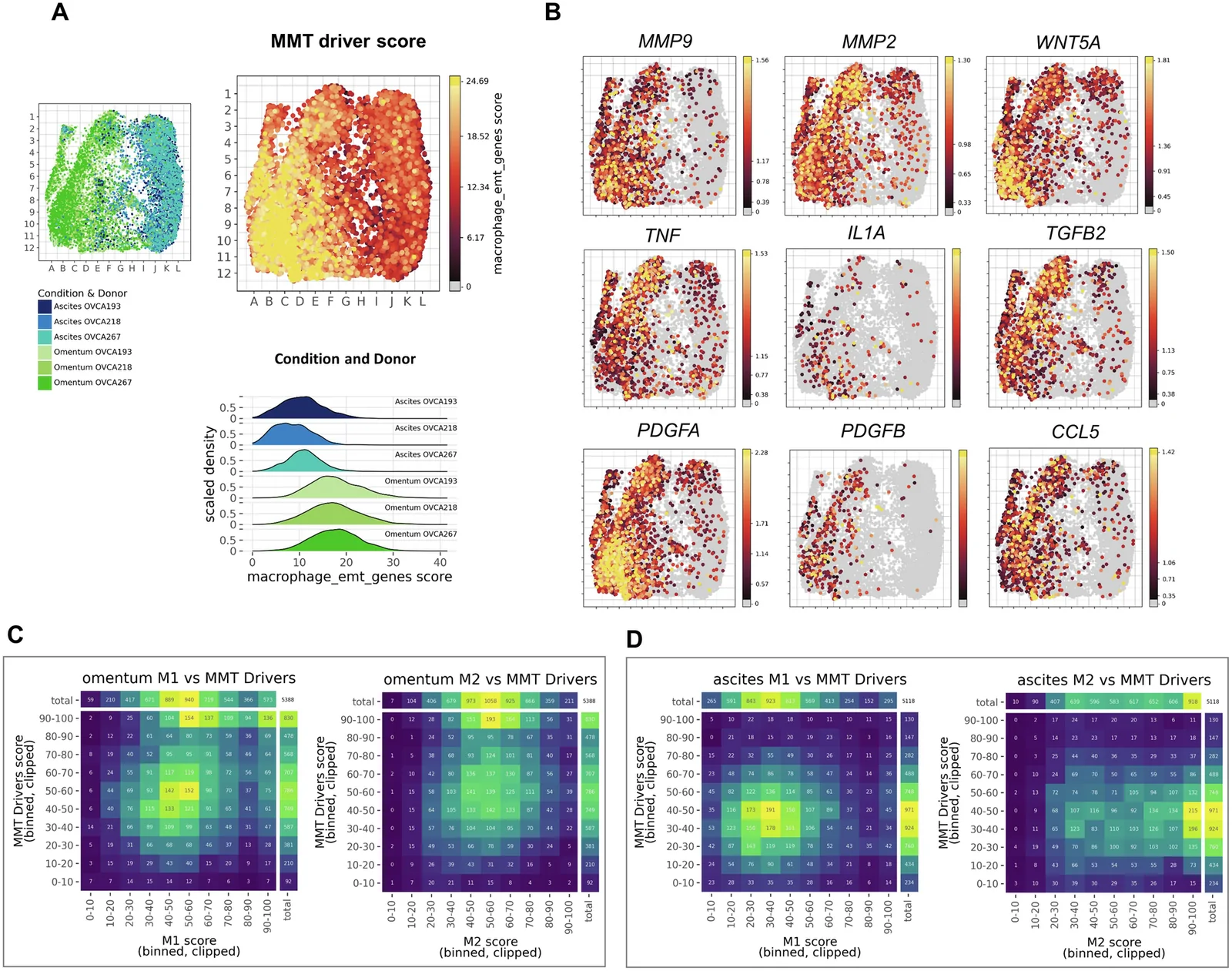
MMT driver gene expression in TAM clusters from omental metastases from OC patients. **A** *Left****:*** Harmony-integrated UMAP depicting scRNA-seq data of TAM derived from matched pairs of ascites and omentum of three OC patients (OVCA193, 218, 267) colored by patient and cell source (as in Fig. 1A). *Right, upper panel****:*** UMAP projection colored by MMT driver score as defined by expression of selected EMT-associated macrophage genes (Supplementary Table S1). Scores are calculated as sum of (normalized, log2 transformed) genes. The color bar is clipped at the 95^th^ percentile. *Right, lower panel*: Density estimate of MMT driver score per patients and cell source. **B** Exemplary UMAPs highlighting the omTAM-specific expression of single genes (*MMP9, MMP2, WNT5A, TNF, IL1A, TGFB2, PDGFA, PDGFB, CCL5*: Subset of the MMT driver score normalized to 10k reads and log2 transformed, color bar clipped at the 95^th^ percentile). **C** 2D heatmap for quantification of overlapping M1 / MMT driver score expression (*Left)* and overlapping M2 / MMT driver score expression (*Right)* in TAM from omentum. **D** 2D heatmap for M1/MMT driver and M2/MMT driver coexpression (as in Fig. 2C) for TAM from ascites. Sum of normalized, log2 transformed expression values are clipped at 95% percentile of all cells and binned by percentile. 2D histograms comprise cell numbers and color scale. The total values are given on the edges. Bin thresholds are constant across panels.

Given the limited understanding of pro-inflammatory M1-like TAMs, we next examined their potential involvement in MESO reprogramming by MMT, as it has been reported that inflammation is associated with the induction of MMT [20]. Analysis of the scRNA-seq dataset using a MMT driver score, composed of genes coding for secreted factors linked to the induction of EMT (Supplementary Table S1), revealed significantly higher expression in omTAM subsets as compared to ascTAMs (Fig. 2A). This pattern was confirmed at the single-gene level for canonical MMT-associated factors, including *MMP2, MMP9, WNT5A, TNF, IL1A, TGFB2, PDGFA/B* and *CCL5* (Fig. 2B). Co-expression analysis further demonstrated that high MMT driver activity was largely confined to M1^+^/M2^+^ omTAM subsets (Fig. 2C), whereas ascTAMs lacked a defined MMT-associated profile (Fig. 2D). These findings suggest a distinct, metastasis-associated TAM population with potential MMT-inducing capacity.

### Pro-inflammatory macrophages induce mesenchymal reprogramming of mesothelial cells

**Fig. 3:**
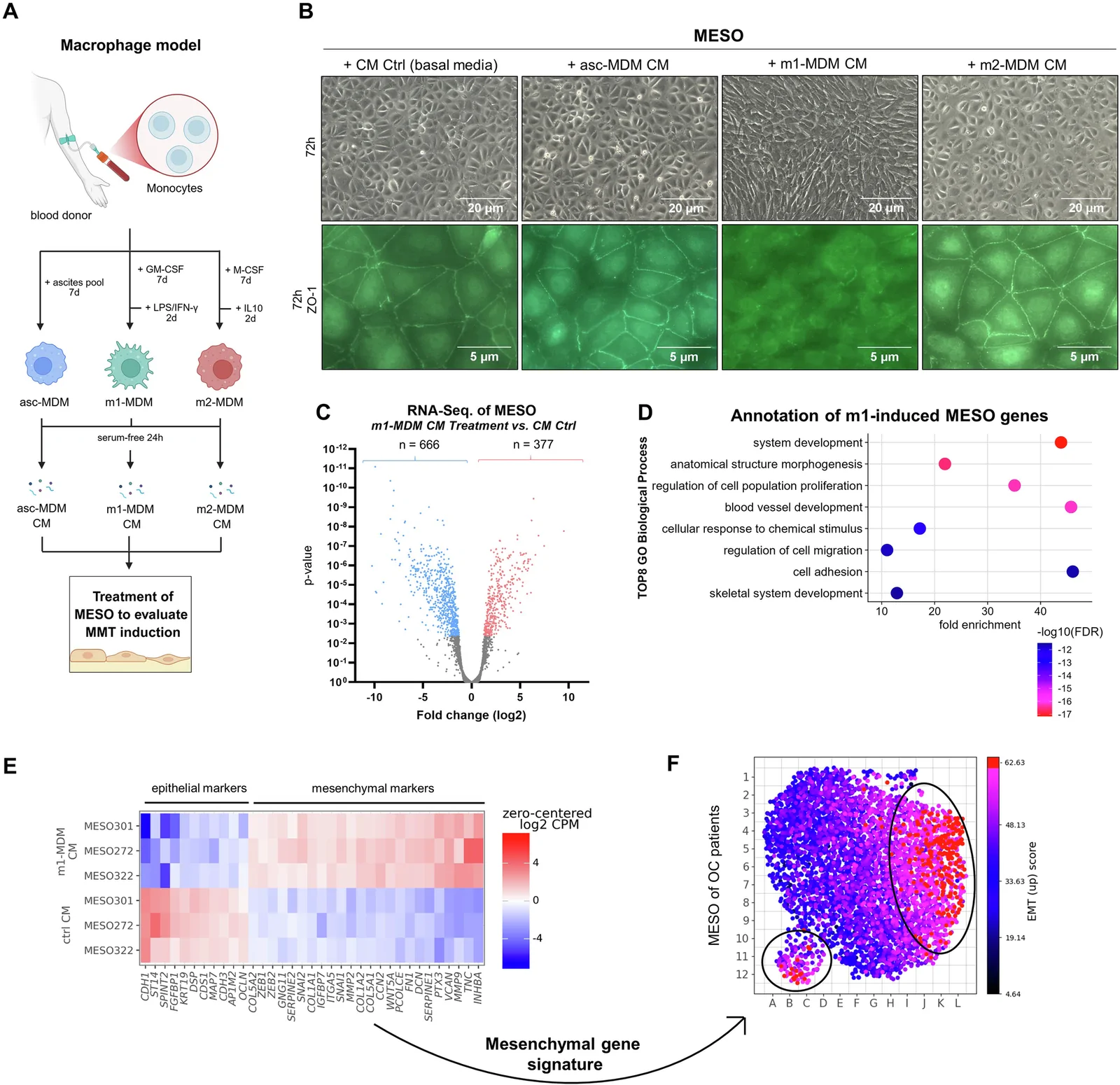
Evaluation of MMT induction in MESO after stimulation with m1-MDM secretome. **A** Experimental macrophage model: Monocytes isolated from PBMC of healthy donors were differentiated in MDM using cell-free ascites (asc-MDM), GM-CSF + LPS/IFNγ (m1-MDM) or M-CSF + IL-10 (m2-MDM), and subsequently cultured for 24 h in serum-free medium to generate CM for MESO treatment. Created in BioRender. Heidemann, S. (2026) https://BioRender.com/aeu6yd0. **B** Morphological changes were microscopically evaluated in MESO treated with CM from asc-MDM, m1-MDM, m2-MDM and Ctrl CM for 72 h (*upper row:* 10x magnification. Scale bar = 20 µm). ZO-1 expression in MESO was visualized by immunofluorescent staining (green) under each condition (*lower row:* 40x magnification. Scale bar = 5 µm). **C** Transcriptional analysis of MESO (*n* = 3 patients) was performed by RNA-seq after exposure to CM from m1-MDM and Ctrl CM for 72 h. The volcano blot illustrates the fold change (FC; log2) induced by m1-MDM: Down-regulated genes in blue (*n* = 666; FDR < 0.05), up-regulated genes in red (*n* = 377, FDR < 0.05), total genes *n* = 11860, non-significantly affected genes *n* = 10817. **D** Functional Annotation of m1-MDM upregulated genes in MESO (as in Fig. 3C) using the “Biological Process Complete” function of Gene Ontology Resource [71]. The plot depicts the top 8 non-redundant terms with fold enrichment sorted by FDR. **E** Heatmap showing the expression levels (adjusted RNA-seq, log2 CPM values) of differentially expressed genes (m1-MDM CM versus CM Ctrl) for MESO from 3 different patients (MESO301, 272, 322) selected for the terms ‘secreted’ and ‘EMT-related’ in the GeneCards database. Epithelial and mesenchymal marker genes are labeled on top. **F** UMAP projection of a previously published scRNA-seq dataset of patient-derived MESO [35] colored by sum normed log2 expression of mesenchymal genes differentially upregulated by m1-MDM CM as in Fig. 3E. Gene lists of the mesenchymal gene signature are provided in Supplementary Table 3. Circled areas indicate MESO clusters with high EMT/MMT score expression.

To assess whether macrophages contribute to mesothelial transdifferentiation, primary MESO derived from OC patients were cultured with CM from MDMs polarized to m1-MDM (GM-CSF/LPS/IFNγ), m2-MDM (M-CSF/IL-10), or ascites-conditioned (asc-MDM) states (Fig. 3A). Exposure to m1-MDM CM induced a pronounced morphological transition in MESO towards an elongated, fibroblast-like phenotype, accompanied by reduced membrane-associated ZO-1 expression. In contrast, MESO morphology remained unchanged upon treatment with m2-MDM, asc-MDM, or CM Ctrl (Fig. 3B). Moreover, we confirmed these findings by a Transwell model of MESO coculture with differentiated m1-, m2- and asc-MDM to allow for dynamic crosstalk via secreted mediators (Supplementary Fig. S2 A). Viability of macrophages, especially m1- and asc-MDM, declines in coculture over time limiting the suitability of the model (Supplementary Fig. S2 B).

Bulk RNA sequencing of MESO cells treated with m1-MDM CM revealed 377 upregulated and 666 downregulated genes as compared to CM Ctrl (Fig. 3C). Upregulated genes were enriched in pathways related to vascular development, proliferation, angiogenesis, migration and adhesion (Fig. 3D). Notably, 35 differentially expressed genes were EMT-associated, including 24 upregulated mesenchymal genes (Fig. 3E), consistent with the observed phenotypic transition in MESO treated with m1-MDM CM. Application of this mesenchymal gene signature to an independent scRNA-seq dataset of ex vivo peritoneal MESO from OC patients [35] identified two MESO clusters with a mesenchymal phenotype (Fig. 3F), supporting the in vivo relevance of these findings.

**Fig. 4:**
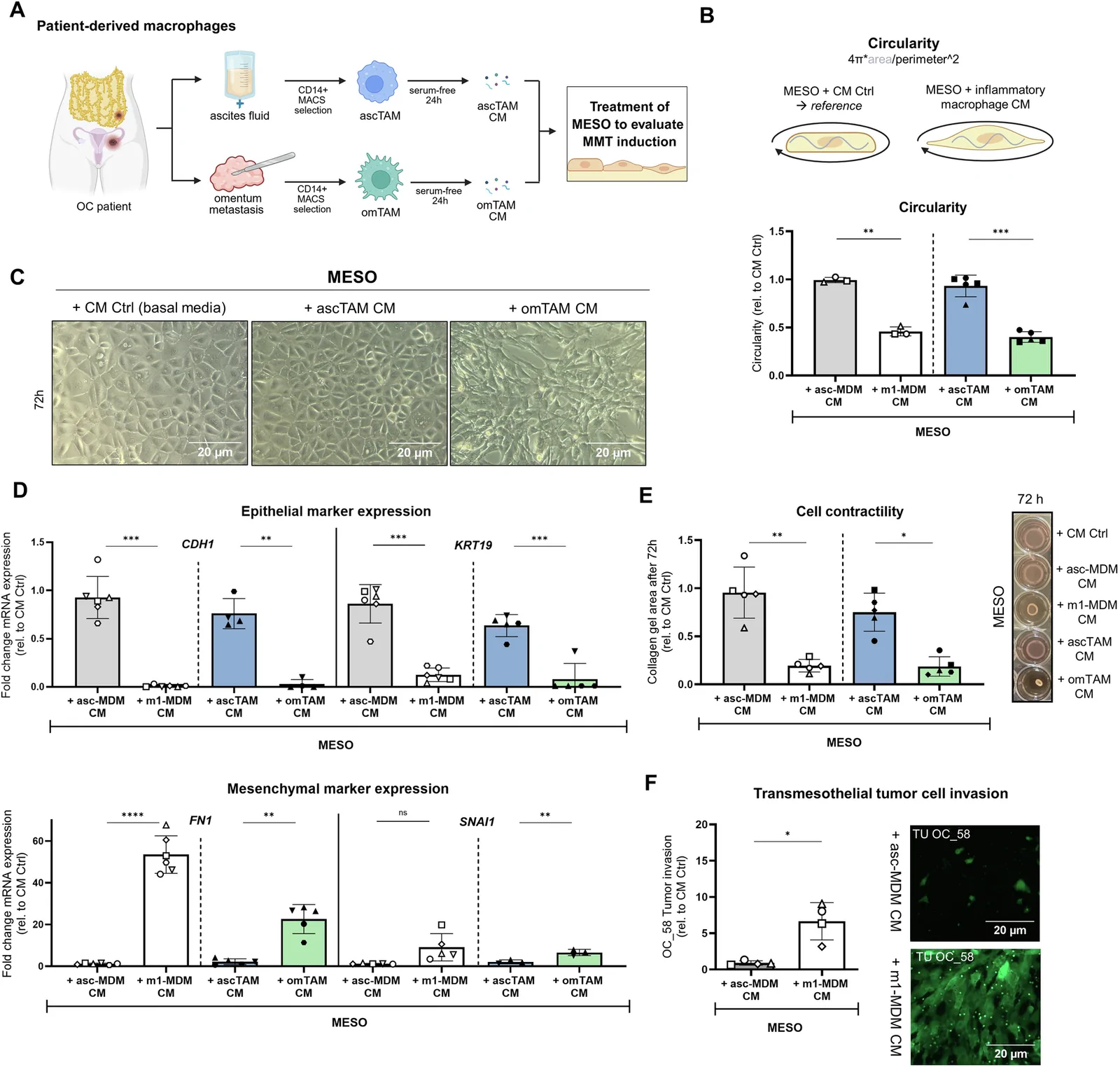
Analysis of phenotypic and function changes of MESO induced by TAM from OC patients. **A** Patient derived macrophages were purified from omentum and ascites by CD14 + MACS selection. CM were collected after 24 h culture in serum-free medium and subsequently used for treatment of MESO (m1-/asc-MDM model see Fig. 3A). Created in BioRender. Heidemann, S. (2026) https://BioRender.com/aeu6yd0. **B** Changes of cell shape were detected microscopically after exposure to matched pairs of omTAM and ascTAM CM of five patients versus Ctrl CM for 72 h. The circularity of 10 cells per visual field (three random visual fields for each condition: *n* = 30 cell total) was analyzed using ImageJ and expressed relative to CM Ctrl. Created in BioRender. Heidemann, S. (2026) https://BioRender.com/aeu6yd0. **C** Representative microscopic pictures of MESO treated with CM of omTAM, ascTAM and Ctrl. Scale bar = 20 µm. **D** Epithelial and mesenchymal marker gene expression were analyzed by RTq-PCR in MESO treated for 72 h with CM from matched pairs of asc-MDM and m1-MDM (*n* = 6) and matched patients’ ascTAM and omTAM (*n* = 3-5). *Upper panel*: *CDH1* and *KRT13* expression. *Lower panel*: *FN1* and *SNAI1* expression. **E** Collagen contraction by MESO. MESO treated for 72 h with CM from asc-MDM and m1-MDM *n* = 5 matched pairs) or patients’ ascTAM and omTAM (*n* = 5 matched pairs) were embedded in collagen gels. Pictures of gel shrinkage were taken after 72 h, gel area of each condition was measured with ImageJ, and normalized to the area of CM Ctrl. Exemplary images are presented. **F** Transmesothelial tumor invasion in collagen matrix. MESO treated with CM of m1-MDM, asc-MDM and Ctrl CM (*n* = 4 donors) were allowed to grow on collagen matrix for 4 d to reach confluence before the primary OCMI tumor cells OC_58 labeled with CellTracker green were applied. Cell invasion was monitored after 24 h using 5% FCS as chemoattractant. Tumor invasion through MDM-CM-pretreated MESO is expressed relative to the CM Ctrl stimulation. Representative pictures of invading tumor cells (green) through MESO stimulated with asc-MDM and m1-MDM CM are shown (Scale bar = 20 µm). Bar plots show the mean ± SD. *p < 0.05, **p < 0.01, ***p < 0.001, ****p < 0.0001 were determined by two-sided, paired t-test; ns: not significant. Paired experiments are indicated through the same symbol. Dotted line (**B**, **D**, **E**) separates data from the MDM model from patients’ TAM.

We therefore postulated that critical MMT-mediators are produced by proinflammatory M1-like TAM in the OC TME leading to a mesenchymal transdifferentiation of adjacent MESO. To test this, we established an experimental setup where CM generated from matched pairs of patient-derived ascTAM and omTAM were applied to primary MESO cultures (Fig. 4A) in addition to the above-mentioned MDM model (Fig. 3A). Only omTAM-derived CM recapitulated the morphological and transcriptional changes induced by m1-MDM CM, namely downregulation of epithelial markers (*CDH1, KRT19*) and upregulation of mesenchymal markers (*FN1, SNAI1*) (Fig. 4B–D). Functionally, omTAM CM induced collagen contraction in MESO, a feature not observed with ascTAM or asc-MDM CM (Fig. 4E). Moreover, MESO reprogramming facilitated transmesothelial invasion of primary OC cells into a collagen matrix (Fig. 4F), indicating a functional role in tumor dissemination.

### TGFβ and ERK/p38 MAPK signaling mediate MMT by pro-inflammatory macrophages

**Fig. 5:**
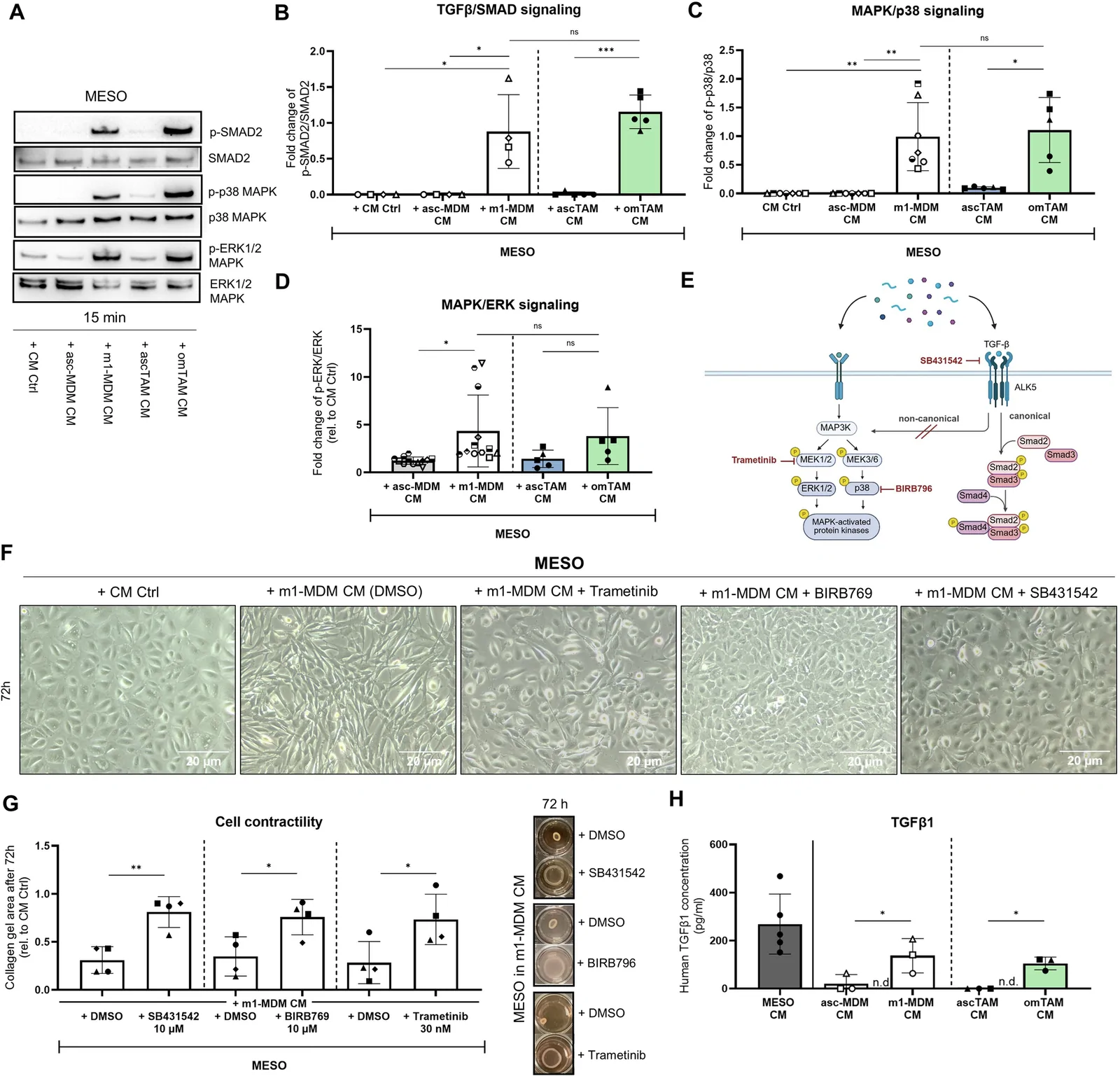
Activation of signaling pathways in MESO by proinflammatory macrophages. **A–D** Detection of SMAD2, p38 and ERK activation in MESO exposed to CM of asc-MDM, m1-MDM, patients’ TAM (om/ascTAM) and Ctrl for 15 min. **A** Exemplary immunoblot pictures are presented. Fold change of phosphorylated relative to total protein is quantified for (**B**) SMAD (*n* = 4 for matched pairs of m1-/asc-MDM; *n* = 5 for matched pairs of asc/om TAM), (**C**) p38 (*n* = 7 for MDM; *n* = 5 for TAM) and (**D**) ERK (*n* = 12 for MDM; *n* = 5 for TAM). **E** Illustration of the ERK/p38 MAPK and TGFβ/SMAD pathways. Inhibitors used in the following experiments are indicated. Created in BioRender. Heidemann, S. (2026) https://BioRender.com/aeu6yd0. **F** Representative microscopic picture of MESO after 72 h exposure to m1-MDM CM with inhibitors trametinib, BIRB769, SB431542 or solvent control (DMSO) compared to CM Ctrl. Scale bar = 20 µm. **G** Collagen contraction of MESO pretreated with m1-MDM CM with inhibitors for 72 h, as above (*n* = 4 donors). Collagen contraction was perform as described in legend of Fig. 4E. Exemplary pictures of collagen gels under different conditions are shown. **H** TGFβ1 level in CM of MESO (*n* = 5 patients), asc-MDM / m1-MDM (matched pairs of *n* = 3 donors) and ascTAM / omTAM (matched pairs of *n* = 3 patients) is determined by ELISA. Bar plots show the mean ± SD. Dotted line separates data from the MDM model from patients’ TAM (**B**–**D**, **H**) or different inhibitor conditions (**G**). Comparisons between conditions in the same donor/patient were performed using a two-sided paired Student’s *t*-test. For datasets in which the control condition was defined as 0, statistical significance relative to control was assessed using a two-sided one-sample *t*-test (**B**–**C**). Comparison between omTAM and m1-MDM CM from different donor / patients was performed by unpaired *t*-test and revealed no significant differences of pSMAD2, p-p38 MAPK and p-ERK levels between omTAM and m1-MDM CM (**B**–**D**). *p < 0.05, **p < 0.01, ***p < 0.001, ns: not significant, n.d.: not detectable.

To identify the signaling pathways underlying macrophage-induced MMT, we focused on TGFβ and MAPK signaling. MESO cells treated with m1-MDM or omTAM CM exhibited robust SMAD activation, whereas asc-MDM and ascTAM CM had no effect (Fig. 5A,B, Supplementary Fig S3 A for additional patients and donors). Concurrently, ERK and p38 MAPK pathways were strongly activated under m1-MDM/omTAM conditions (Fig. 5A,C,D, Supplementary Fig S3 A). Pharmacological inhibition of MEK1/2 (trametinib) or p38 (BIRB796) effectively suppressed MAPK activation, while TGF-β receptor inhibition (SB431542) selectively blocked SMAD phosphorylation without affecting ERK/p38 signaling (Supplementary Fig. S3 B–F), indicating parallel pathway activation by rather different MMT drivers in the macrophage secretome as outlined in Fig. 5E. Functionally, pharmacological inhibition of TGFβ, ERK, or p38 signaling partially reversed m1-MDM-induced mesenchymal morphology and reduced *FN1* expression, with the strongest effect observed for TGFβ blockade (Fig. 5F; Supplementary Fig. S3 G). Collagen contraction was likewise attenuated by these inhibitors (Fig. 5G). The observed effects were not associated with inhibitor cytotoxicity as shown by cell viability staining (Supplementary Fig. S3 H). Thus, we concluded that TGFβ signaling next to the ERK/p38 MAPK pathway seems to play a major role in MMT induction by pro-inflammatory macrophages.

ELISA and RT-qPCR analyses confirmed elevated TGFβ1 and *TGFB2* expression in pro-inflammatory macrophages, particularly in omTAMs (Fig. 5H; Supplementary Fig. S3 I). Notably, MESO cells themselves represented the primary source of TGFβ1 within the TME according to our previously published transcriptomic data [17], which was confirmed by ELISA (Fig. 5H). We therefore assumed that macrophage-derived factors in the OC TME amplify endogenous TGFβ signaling in MESO.

### IL-1α is a key macrophage-derived driver of MMT

**Fig. 6:**
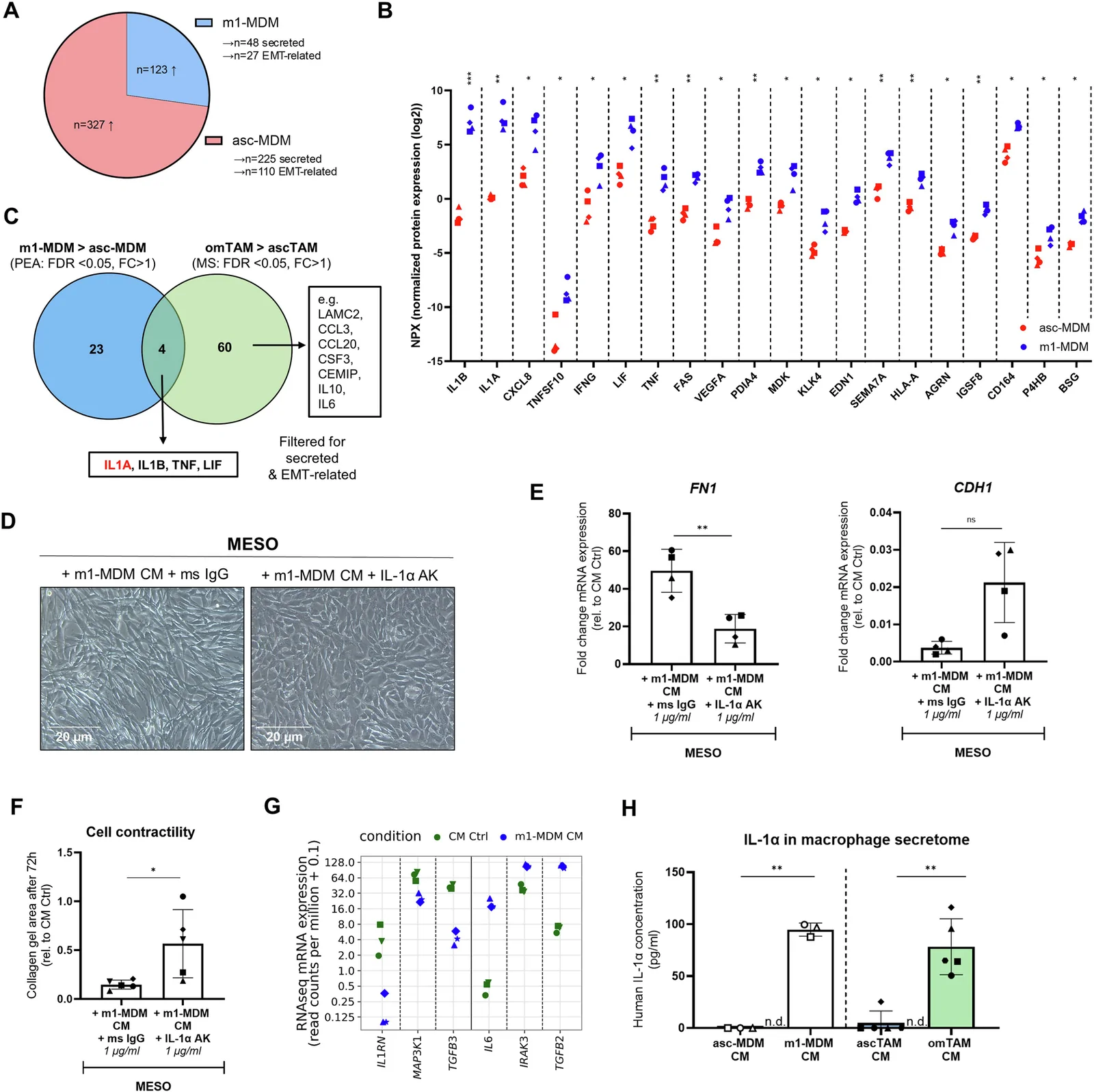
Identification of IL-1α as central MMT-drivers in the proinflammatory macrophage secretome. **A–B** PEA-based affinity proteomics of m1-MDM compared to asc-MDM secretomes from *n* = 3 donors. **A** The pie-chart illustrates the distribution of upregulated proteins in asc-MDM and m1-MDM CM including numbers of proteins annotated as ‘secreted’ and ’EMT-related’ according to in the genecards.org database (FDR < 0.05 and FC > 1). **B** The dot plot depicts the signals for TOP 20 EMT-related proteins upregulated in m1-MDM versus asc-MDM CM (FDR < 0.05 and FC > 1, determined by paired *t*-test), sorted by FC. Results are expressed as normalized protein expression (NPX, log2). *FDR < 0.05, **FDR < 0.01, *** FDR < 0.001. **C** The Venn diagram depicts the overlap between upregulated proteins in m1-MDM CM versus asc-MDM CM as detected by PEA-based proteomics (**A**, **B**) and upregulated proteins in omTAM versus matched ascTAM (*n* = 6 OC patients) based on mass-spectrometry analysis (FDR < 0.05 and FC > 1, determined by paired t-test; Supplementary Table S6). Both protein sets were filtered for ‘secreted’ and ‘EMT-related’ (as described above), resulting in the identification of 4 proteins upregulated in both m1-MDM and omTAM. **D** Representative microscopic pictures showing the morphological changes in MESO exposed to m1-MDM CM blocked by neutralizing IL-1α Ab compared to a ms IgG control for 72 h. Scale bar = 20 µm. **E** Mesenchymal (FN1, *left*) and epithelial (CDH1, *right*) gene expression were analyzed by RTq-PCR in MESO treated for 72 h with m1-MDM CM and IL-1α Ab or ms IgG control (*n* = 4). **F** Collagen contractility was determined in MESO pretreated for 72 h with m1-MDM CM and IL-1α Ab or ms IgG control (*n* = 5) as described in legend of Fig. 4E. **G** IL1-signaling and target genes derived from the MSigDB C2 gene set WP_SIGNAL_TRANSDUCTION_THROUGH_IL1R showing significant differential expression in the RNA-seq data of MESO treated with m1-MDM CM compared to CM Ctrl (as in Fig. 3C). *Left hand side*: Genes expressed higher in control condition. *Right hand side*: Genes expressed higher in the ‘m1-MDM CM’ condition. **H** IL-1α concentrations in CM of asc-MDM / m1-MDM (*n* = 3 donors) and ascTAM / omTAM (*n* = 5 patients) are determined by ELISA. Dotted line separates data from the MDM model from patients’ TAM. Plots show the mean ± SD. Comparison of conditions was performed by two-sided, paired *t*-test. *p < 0.05, **p < 0.01, ns: not significant, n.d.: not detectable.

To identify additional MMT-inducing factors, we performed affinity-based proteomic profiling of m1-MDM versus asc-MDM secretomes. Among 123 upregulated proteins in m1-MDM CM (FDR < 0.05), 27 were classified as secreted and EMT-associated, including many cytokines (Fig. 6A; TOP 20 upregulated EMT-related proteins shown in Fig. 6B). Functional screening was performed with a panel of EMT-related candidate cytokines (IL-1α, IL-1β, IFNγ, LIF, TNFα, VEGFA, MDK, EDN1, SEMA7A) excluding TNFSF10/TRAIL and FAS which are mainly related to death receptor pathways, as well as CXCL8 due to the lack of receptor expression on MESO. Our analysis identified IL-1α, IL-1β, TNFα and to a lesser extent IFNγ, as capable of inducing partial morphological changes in MESO cells, when applied as recombinant proteins (Supplementary Fig. S4 A-E). Notably, IL-1α, IL-1β, and TNFα were also found among the significantly upregulated proteins in patient-derived omTAMs, based on a complementary mass spectrometry (MS) based proteomics dataset of matched omTAM and ascTAM pairs from six OC patients (Fig. 6C). However, none of these mediators alone or combined reproduced the full MMT phenotype or affected the collagen contractility of MESO (Supplementary Fig. S4 F), suggesting cooperative functions with other factors yet unknown. Possible candidates in this context were identified from MS data comparing omTAM and ascTAM secretomes; these include several additional EMT-linked proteins – such as LAMC2, CCL3, CCL20, CSF3, CEMIP, IL-10, and IL-6 – which were not significantly upregulated in m1-MDMs, but may contribute to MMT regulation in a clinical context.

Remarkably, only neutralization of IL-1α in m1-MDM CM significantly attenuated morphological transformation, mesenchymal gene expression and collagen contraction in MESO cells (Fig. 6D–F), whereas IL-1β or TNFα blockade had no effect (Supplementary Fig. S5 A–D). IL-1α specifically mediated ERK/p38 activation but did not influence SMAD signaling as demonstrated by the use of recombinant IL-1α or a neutralizing IL-1α antibody approach (Supplementary Fig. S5 E–G). Transcriptomic analysis further revealed activation of IL-1 signaling pathways in MESO by proinflammatory macrophages, including upregulation of *IL6, IRAK3* and *TGFB2* and downregulation of IL-1 receptor antagonist *IL1RN* (Fig. 6G).

**Fig. 7:**
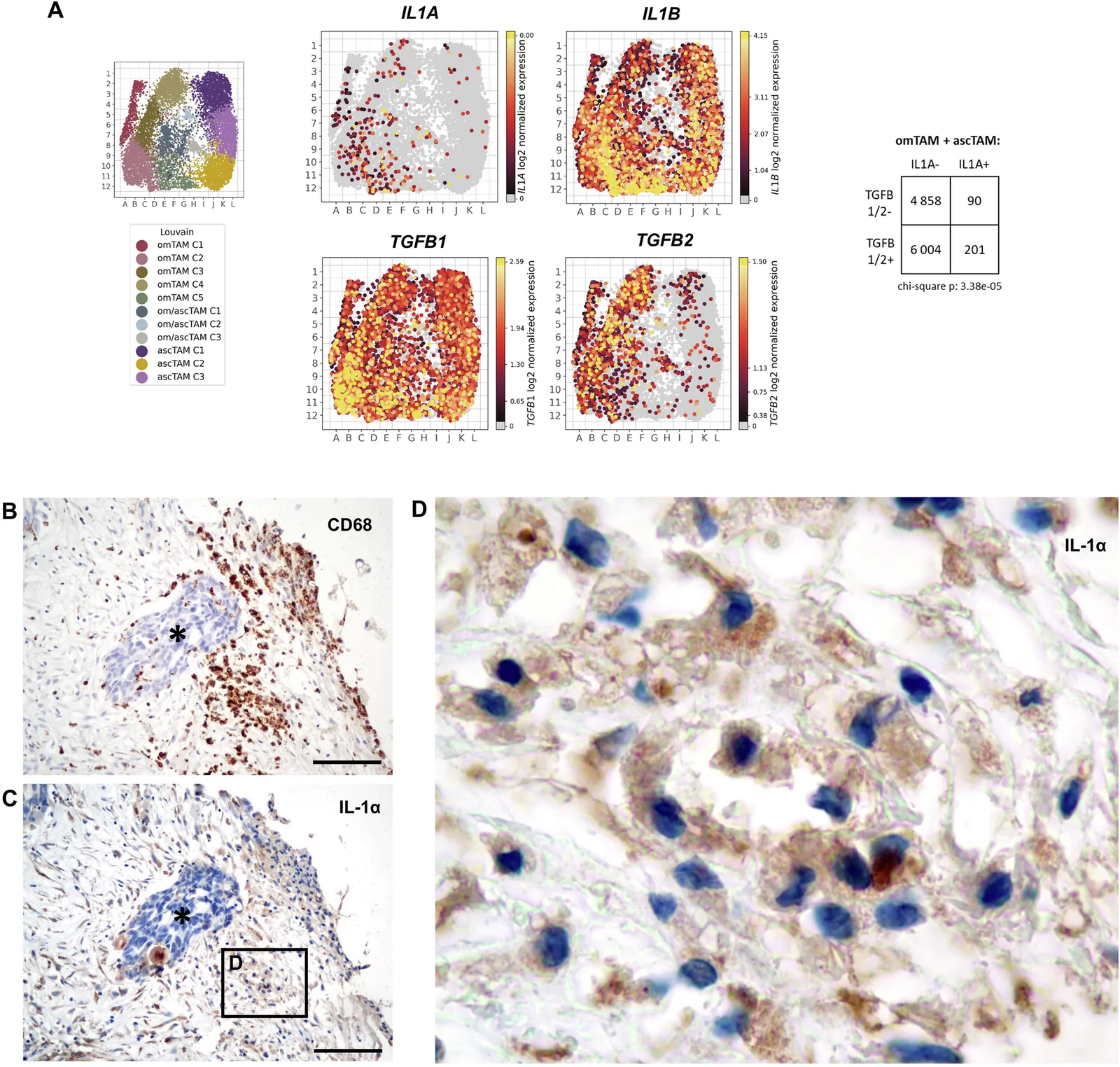
Validation of IL-1α^+^ macrophages in peritoneal metastases of high-grade OC patients. **A** IL1A and TGFB1/2 gene expression in ascTAM and omTAM subsets of OC patients on single cell transcriptomic level. Harmony-integrated UMAP projection depicting scRNA-seq data of TAM derived from matched pairs of ascites and omentum of three OC patients (OVCA193, 218, 267) colored by patient and cell source (as in Fig. 1). *IL1A, IL1B, TGFB1* and *TGFB2* expression in TAM cluster is colored by gene expression (normalized to 10k reads and log2 transformed). Coexpression of *IL1A* and *TGFB1/2* genes in omTAM and ascTAM is analyzed by chi-square test. **B–D** Immunohistochemistry of IL1α^+^ macrophages adjacent to metastases in a clinical specimen of high-grade OC. **B** CD68 stain demarking peritoneal macrophages neighboring an invasive tumor cell cluster (*) of high-grade serous OC in paraffin sections. **C**, **D** Immunohistochemical staining for IL-1α reveals granular IL-1α expression in these macrophages. Staining was performed on serial paraffin sections. Scale bar, 100 µm.

Consistent with these findings, IL-1α secretion was significantly higher in omTAMs as compared to ascTAMs from OC patients and already observed in m1-MDM / asc-MDM model (Fig. 6H). IL-1β and TNFα secretion levels follow the same trend, but with a broader deviation in the m1-MDM and patient-derived TAM subsets (Supplementary Fig. S5 H,I). Moreover, scRNA-seq analysis localized *IL1A* expression predominantly to omTAM clusters (Fig. 7A) showing a broad overlap with high MMT-driver score expression (Fig. 2A). This also applies to *TGFB2*, whereas *IL1B* and *TGFB1* gene expression is widely distributed among omTAM and ascTAM subsets (Fig. 7A). Co-expression analysis demonstrated overlap between *IL1A* and *TGFB1/2* expression at the single-cell level, suggesting coordinated cytokine production in patients’ TAMs. Importantly, immunohistochemical analysis confirmed the presence of CD68⁺/IL-1α⁺ macrophage clusters in proximity to peritoneal micrometastatic lesions of high-grade OC (Fig. 7B–D). In summary, these data support the idea that IL-1α released by pro-inflammatory macrophage together with TGFβ jointly act as critical MMT drivers supporting tumor implantation during early steps of metastasis, whereas the function of IL-1β and TNFα in this context is less clear.

### IL-1α amplifies MMT via induction of autocrine TGFβ signaling in MESO

**Fig. 8:**
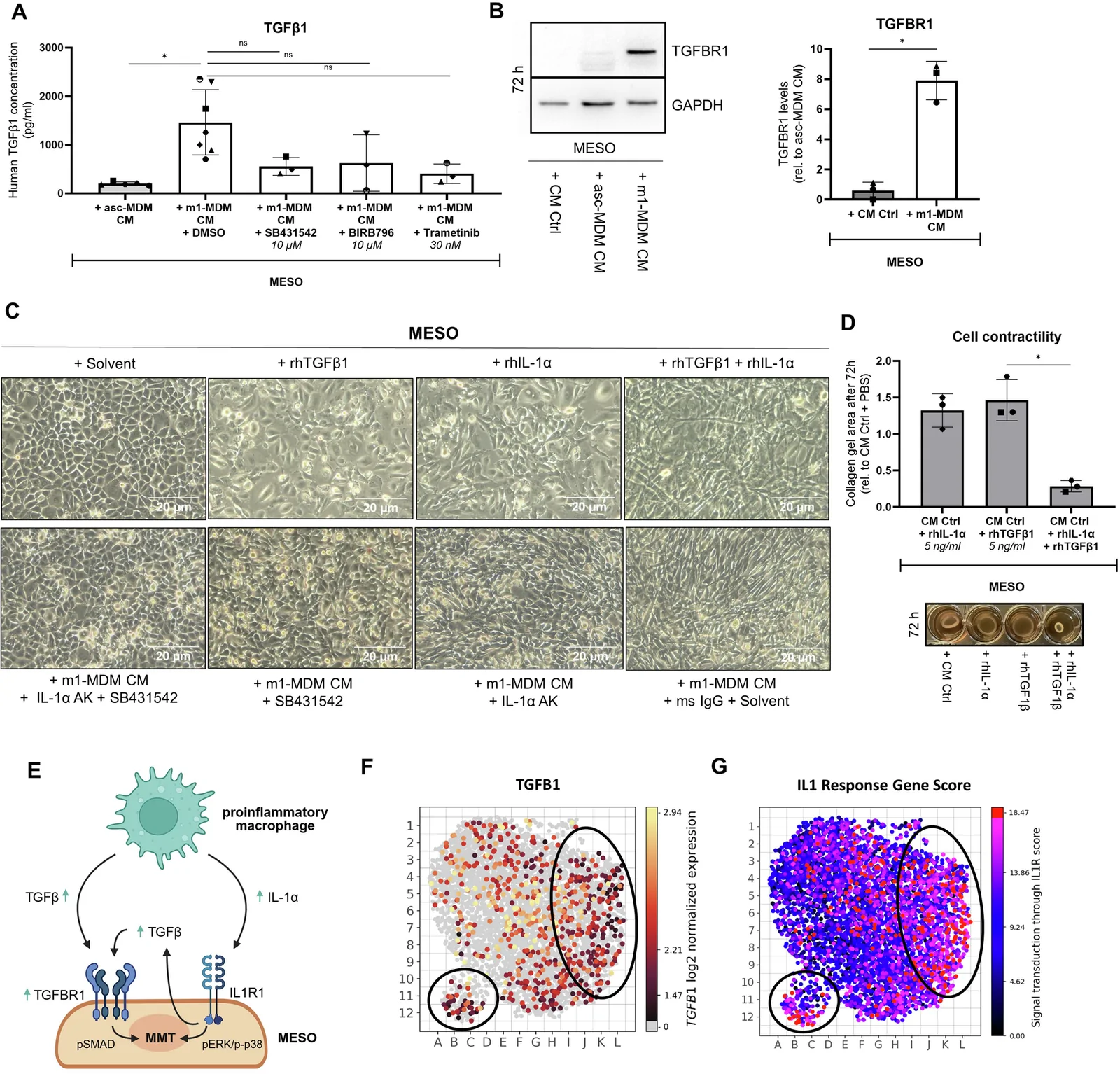
IL-1α-mediated upregulation of autocrine TGFβ expression in MESO amplifies MMT. **A** TGFβ1 concentrations in CM of MESO treated with m1-MDM CM + inhibitors (SB413162, BIRB798, trametinib) or + DMSO (solvent control) (*n* = 3 donors for each condition) for 72 h as determined by ELISA. Treatment with asc-MDM CM was included as a control for m1-MDM CM (*n* = 5 matched pairs). **B** Immunoblot of TGFBR1 expression in MESO treated with m1-MDM CM, asc-MDM CM or CM Ctrl for 72 h. An exemplary blot of TGFBR1 expression in MESO is shown, as well as the quantification (*n* = 3) relative to the asc-MDM CM condition. **C** Representative microscopic pictures of MESO stimulated with rhTGFβ1 and rhIL-1α (alone or combined), and after exposure to m1-MDM CM + TGFβ inhibitor SB431542 and neutralizing IL-1α Ab (alone or combined) for 72 h. Solvent and ms IgG were included as controls. Scale bar = 20 µm. **D** Collagen contractility was determined in MESO pretreated for 72 h with rhTGFβ1 and rhIL-1α (alone or combined) (*n* = 3) as described in legend of Fig. 4E. Plots show the mean ± SD. Multiple-group-comparison were analyzed with ANOVA (one-way) and Tukey’s post hoc test (**A**, **D**) or two-sided, paired *t* test (Fig. 8B). *p < 0.05, ns: not significant. **E** Schematic illustration of proposed mechanism of IL-1α-mediated upregulation of autocrine TGFβ expression in MESO. Created in BioRender. Heidemann, S. (2026) https://BioRender.com/aeu6yd0. **F**, **G** UMAP projections of a previously published scRNA-seq dataset of patient-derived MESO [35] colored by (**F**) normed log2 expression of *TGFB1* or (**G**) by sum normed log2 expression of ILR1-related genes differential in m1-MDM CM-treated MESO compared to CM Ctrl as in Fig. 6G. Gene lists of the IL1 response gene score are provided in Supplementary Table 3. Circled areas indicate MESO clusters with high EMT/MMT score expression derived from Fig. 3F.

Given the known regulatory link between IL-1α and TGFβ [36, 37], we investigated whether IL-1α enhances MMT by promoting autocrine TGFβ signaling in MESO. Treatment with m1-MDM CM significantly increased TGFβ1 secretion in MESO, an effect partially recapitulated by recombinant IL-1α (Fig. 8A; Supplementary Fig. S6A). IL-1α neutralization, by contrast, attenuated m1-MDM-induced *TGFB1/2* gene expression (Supplementary Fig. S6 B,C). Pharmacological inhibition of TGFβ or ERK/p38 MAPK signaling also reduced TGFβ1 expression as shown by ELISA (Fig. 8A), indicating pathway interdependence. Additionally, m1-MDM CM upregulated TGFBR1 expression, enhancing MESO responsiveness to TGFβ (Fig. 8B).

As a proof-of-principle for the cooperative activity of IL-1α and TGFβ in MMT induction, combined stimulation with IL-1α and TGFβ fully reproduced the MMT phenotype, whereas dual inhibition of IL-1α and TGFβ signaling completely abrogated macrophage-induced MMT (Fig. 8C). These morphological findings are further emphasized by the evaluation of the mesenchymal marker *FN1* under these experimental conditions (Supplementary Fig. S6 D). Functional assays confirmed that only the combination of both cytokines induced collagen contractility as comparable to that mediated by pro-inflammatory macrophages (Fig. 8D).

Collectively, these data indicate that IL-1α derived from pro-inflammatory macrophages amplifies TGFβ-mediated MMT by inducing autocrine TGFβ production and enhancing receptor expression in MESO by upregulation of TGFBR1 expression thereby driving mesothelial reprogramming and facilitating early metastatic progression in OC (Fig. 8E). The physiological relevance of this mechanism is further supported by analysis of an independent scRNA-seq dataset of ex vivo peritoneal MESO from OC patients [35] showing partially overlapping *TGFB1* (Fig. 8F) and IL-1 response gene score expression (Fig. 8G, Supplementary Table S1) in two MESO clusters with a mesenchymal phenotype (as in Fig. 3F).

## Discussion

Reciprocal interactions between macrophages and stromal, immune and tumor cells are key determinants of a pro-tumorigenic and immunosuppressive TME. While M2-like TAM states are widely recognized as major drivers of tumor progression through promotion of invasion, extracellular matrix remodeling, angiogenesis and immune evasion [4], the role of M1-like pro-inflammatory TAM subsets remains less clearly defined. In this study, we demonstrate a compartment-specific reprogramming of TAMs in metastatic OC, characterized by the emergence of a prominent M1^+^/M2^+^ mixed phenotype in omental metastases. This observation challenges the classical binary M1/M2 framework and supports the concept of a functional continuum of TAM polarization states in vivo. Importantly, we identify a previously unrecognized function of these pro-inflammatory omTAM subsets in promoting MMT, primarily via IL-1α secretion and subsequent activation of a TGFβ–dependent feedback loop. Functionally, this macrophage-driven MMT compromises mesothelial barrier integrity and facilitates transmesothelial tumor invasion in vitro, consistent with histopathological evidence of mesothelial disruption at metastatic sites [28, 38].

MESO are highly plastic and play essential roles in tissue homeostasis, repair and immune surveillance. However, this plasticity may be co-opted within the TME to support tumor dissemination, particularly in cancers such as OC that spread predominantly within the peritoneal cavity. Recent scRNA-seq studies elucidated the existence of different MESO clusters comprising subsets with mesenchymal gene expression signatures in various cancer types (review in [39]). By integrating our experimental findings with published scRNA-seq datasets of patient-derived ex vivo MESO [35], we identified mesothelial subsets with mesenchymal gene expression signatures that closely resemble those induced by pro-inflammatory macrophages in vitro. These transcriptomic observations were supported by functional assays demonstrating that both, M1-polarized macrophages and patient-derived omTAMs, induce comparable morphological, transcriptional as well as functional changes in MESO, including loss of epithelial characteristics, acquisition of fibroblast-like properties and increased contractility. Together, these data support a model in which pro-inflammatory TAMs actively regulate mesothelial plasticity and contribute to the establishment of a permissive premetastatic niche in the peritoneum. Moreover, they validate the m1-MDM model as a functionally relevant surrogate for inflammatory TAM subsets observed in patients.

Mechanistically, our data identify IL-1α as a central macrophage-derived driver of MMT. Although IL-1α and IL-1β share receptor signaling and induce partially overlapping phenotypic changes in MESO when applied as recombinant cytokines [40], only IL-1α neutralization effectively attenuated macrophage-induced MMT, indicating a non-redundant role in this context. This distinction may be explained by the dual function of IL-1α as both a cytokine and an alarmin, capable of initiating and amplifying broader inflammatory signaling cascades [36, 37]. Consistent with this, we observed that IL-1α stimulation enhances endogenous TGFβ production in MESO and upregulates TGFBR1 expression, thereby increasing cellular responsiveness to TGFβ. These findings support a model in which macrophage-derived IL-1α acts upstream of a self-reinforcing TGFβ signaling loop in MESO, amplifying and stabilizing the mesenchymal phenotype. In addition, the co-expression of *IL1A* and *TGFB* isoforms within omTAM subsets suggests that coordinated cytokine production at the single-cell level may further potentiate this process within the metastatic niche.

At the signaling level, we demonstrate that IL-1α predominantly activates ERK and p38 MAPK pathways, whereas TGFβ signaling proceeds via canonical SMAD activation. Both signaling axes contribute to MMT induction and to the regulation of TGFβ expression and receptor availability in MESO. Notably, ERK/p38 activation did not appear to be mediated by non-canonical TGFβ signaling as also reported by others [32], but rather by IL-1α–dependent mechanisms, indicating parallel rather than hierarchical pathway activation. Pharmacological inhibition experiments further revealed that MMT may be effectively suppressed at multiple levels: Blockade of IL-1α or TGFβ signaling resulted in near-complete inhibition of mesenchymal reprogramming, whereas targeting downstream MAPK pathways achieved only partial attenuation. These findings suggest that upstream cytokine signaling represents a more effective intervention point and underscore the cooperative nature of IL-1α and TGFβ signaling in driving MMT.

The observation that MESO themselves represent a major source of TGFβ within the TME further refines this model. Rather than acting solely as passive targets, mesothelial cells actively contribute to the amplification of pro-fibrotic and pro-invasive signaling through autocrine TGFβ production. In this context, IL-1α derived from pro-inflammatory macrophages appears to function as a critical trigger that initiates and sustains this feedback loop. This interplay between exogenous macrophage-derived IL-1α and endogenous MESO-derived TGFβ likely represents a key mechanism underlying mesothelial reprogramming and metastatic niche formation in OC. At the same time, the potential contribution of omTAM subsets co-expressing both IL-1α and TGFβ suggests that multiple, possibly redundant, sources of these cytokines may converge to reinforce MMT in vivo.

From a therapeutic perspective, these findings identify several potential targets for interfering with macrophage-driven mesothelial reprogramming and metastatic progression. Although TGFβ is a central regulator of OC progression, therapy resistance and immune suppression, no approved TGFβ–targeting therapies are currently available in this setting [41]. In contrast, IL-1α-neutralizing antibodies (such as bermekimab) and IL-1 receptor antagonists (such as anakinra), have already entered early-phase clinical evaluation in solid tumors [42]. In light of the clinical importance of IL-1α in OC development and metastasis [43, 44], IL-1–targeting strategies may represent promising candidates for further investigation in OC. Targeting IL-1α may be particularly advantageous, as it acts upstream of TGFβ induction and MAPK activation, thereby potentially disrupting multiple pro-tumorigenic pathways simultaneously. In addition, the MEK1/2 inhibitor trametinib has received much attention in the treatment of low grade OC characterized by a high frequency of activating mutations in the MAPK pathway [45], and beyond that has shown MMT-blocking potential in a mouse model of bowel adhesion formation [46]. MEK inhibition by trametinib, which partially attenuated MMT in our model, may provide a complementary approach by targeting downstream signaling nodes in high-grade OC, although its effects appear less pronounced compared to direct cytokine blockade.

Beyond mesothelial reprogramming, macrophage-derived IL-1α is also assumed to promote EMT in OC cells, thereby enforcing the metastatic process by acting on tumor and stroma cells, both. This aligns with published findings in various solid tumors where IL-1α upregulation drives EMT [47, 48]. Noteworthy, EMT processes in breast cancer cells can be induced by macrophages via secretion of proinflammatory factors (IL-1α among others) [49] and through upregulation of IL-1α expression in the tumor cell [50], which further supports a possible clinical relevance of IL-1α targeting approaches in OC.

Of note, previous clinical studies of IL-1α blockade have not demonstrated conventional anti-tumor efficacy in advanced cancer settings. In metastatic colorectal cancer, bermekimab improved a composite clinical-benefit endpoint without clear evidence of tumor-directed activity [51]. A subsequent phase III study evaluating overall survival was terminated for futility (NCT01767857), and more recent reviews of cytokine-targeted therapies highlight the context dependence of such approaches and suggest that their efficacy may be limited in advanced disease stages [36, 42]. An important distinction between these studies and the context of our findings may therefore lie in the therapeutic objective. Prior trials primarily targeted advanced, refractory disease with the aim of achieving clinical benefit or disease control, whereas our data point to a role of IL-1α in early microenvironmental processes, including mesothelial reprogramming and metastatic niche formation. This suggests that targeting IL-1α may be more relevant in an adjuvant setting following tumor resection, where the goal is to prevent early dissemination and relapse rather than to induce regression of established tumors.

Despite the new insights provided by the present study, several limitations should be considered. The use of in vitro coculture systems cannot fully recapitulate the spatial complexity, cellular diversity and dynamic interactions of TAM subsets within the in vivo TME as already addressed by others [52]. However, this limitation was mitigated by integrating single-cell transcriptomic analyses and validating key findings in patient-derived TAM populations. Furthermore, while our data establish a functional role for IL-1α–producing TAMs in mesothelial reprogramming, their prognostic significance and therapeutic relevance in clinical settings remain to be determined. Future studies should therefore focus on validating these findings in in vivo models of OC metastasis and on assessing whether targeting the IL-1α–TGFβ axis may effectively disrupt metastatic niche formation and improve clinical outcomes.

In summary, our study identifies pro-inflammatory TAM subsets in omental metastases as active regulators of mesothelial plasticity and highlights IL-1α as a key mediator linking inflammation to MMT and metastatic progression. These findings provide mechanistic insight into the early steps of peritoneal dissemination in OC and define a potential therapeutic axis that may be exploited to limit metastatic spread.

## Materials and methods

### Isolation and culture of cells from OC patients

Patients with high-grade OC undergoing primary debulking surgery at the University Hospital Marburg provided ascites and metastatic omental tissue for downstream analyses. None of the patients had received prior treatment. Collection and analysis of human specimens were approved by the local ethics committee (reference no. 205/10), and all donors provided written informed consent in accordance with the Declaration of Helsinki. Patient characteristics are summarized in Supplementary Table S2.

Stable primary tumor cell cultures (OCMI tumor cells) were generated from tumor spheroids isolated from ascites using a modified protocol based on Ince et al [53] and cultured in OCMI medium as previously described [54]. The patient-derived OCMI cell line OC_58 was used for invasion assays.

MESO were isolated from macroscopically tumor-free omental tissue by enzymatic digestion as described previously [17]. Cell purity exceeded 95% CD45⁻/EpCAM⁻ cells as confirmed by flow cytometry, and cells displayed a characteristic cobblestone morphology in culture. MESO were maintained in OCMI medium and used for functional assays up to passage 6.

TAMs were isolated from matched ascites and omental metastases as described previously [17]. Briefly, ascitic mononuclear cells were obtained by Ficoll density gradient centrifugation (Lymphocyte Separation Medium 1077; Capricorn, Ebsdorfergrund, Germany) and ascTAMs were purified with magnetic cell sorting (MACS) using CD14 magnetic microbeads (Miltenyi Biotec, Bergisch Gladbach, Germany). Omental tissue was mechanically dissociated and enzymatically digested with collagenase I and II (370 U/ml; Sigma Aldrich, Taufkirchen, Germany) at a ratio of 10 ml per 5 g tissue for 1 h at 37 °C with agitation. Following filtration through 1000 µm (Pluriselect, Thermo Fisher Scientific, Waltham, MA, USA) and 100 µm meshes (Miltenyi Biotec), omentum-derived TAMs (omTAMs) were purified by CD14⁺ MACS selection following the same procedure as for ascTAM. Immediately after isolation, TAMs were plated at ≥3.1 × 10⁵ cells/cm² and allowed to adhere for 1 h in RPMI-1640 supplemented with 5% human AB serum (Sigma-Aldrich) and 1% sodium pyruvate prior to conditioned media (CM) generation.

### Isolation and differentiation of monocytes from healthy donors

Peripheral blood mononuclear cells (PBMCs) from healthy donors were obtained from leukoreduction system (LRS) chambers provided by the Center for Transfusion Medicine and Hemotherapy (University Hospital Giessen and Marburg). Monocytes were isolated and differentiated as previously described [55]. After Ficoll separation, monocytes were enriched by plastic adherence (1 h) in RPMI-1640 supplemented with 5% human AB serum. For generation of ascites-conditioned macrophages (asc-MDM), monocytes were cultured for 7 days in pooled cell-free ascites (from 10 OC patients) supplemented with 50 ng/ml M-CSF (macrophage CSF, Biolegend, San Diego, CA, USA). Although M-CSF, in contrast to GM-CSF, is already a high-abundance ascites compound in malignant OC ascites ([56] and own data in Supplementary Fig. S7 A), M-CSF was added to the ascites pool to balance for differences in M-CSF levels between individual ascites batches, which may otherwise impact cell viability during macrophage differentiation in vitro. M1-polarized macrophages (m1-MDM) were generated by culturing monocytes in RPMI-1640 containing 5% human AB serum, 1% sodium pyruvate, and 100 ng/ml GM-CSF (Peprotech, Hamburg, Germany) for 5 days, followed by stimulation with 100 ng/ml LPS (Sigma-Aldrich) and 20 ng/ml IFNγ (Biozol, Echingen, Germany). M2 macrophages (m2-MDM) were generated by culturing monocytes in 50 ng/ml M-CSF for 5 days, followed by stimulation with 20 ng/ml IL-10 (Biozol) for 2 days. Representative microscopic pictures of different MDM polarization states are depicted in Supplementary Fig. S7 B.

### Flow cytometric analysis of macrophage phenotypes

The differentiation phenotype of m1-, m2- and asc-MDM was monitored by flow cytometry on a FACS Celesta instrument using Diva Software (BD Biosciences) and analysis by FlowJo™ v10.8 Software (BD Life Sciences) as described previously [14, 55]. Surface expression of classical M1 and M2 marker was detected using the following antibodies: anti-human CD14-FITC (#130-113-146, Miltenyi Biotec), CD86-FITC (#130-113-571, Miltenyi Biotec), CD16-PE-Cy7 (#15588356, eBioscience, Frankfurt, Germany, CD163-PE (#12-1639-42, eBioscience) and HLA-DR-APC (#17-9956-42, eBioscience). Isotype control antibodies were purchased from Miltenyi Biotec and eBioscience. The percentage of stained cells and mean fluorescence intensities (MFI) were calculated. The phenotypic characterization of m1-, m2, and asc-MDM differentiation from different healthy blood donors is presented in Supplementary Fig. S7 C.

### Generation of conditioned media

Differentiated MDM (day 7) and freshly isolated TAMs were washed three times with serum-free medium (M199 mixed 1:2 with DMEM/F-12) (M199: Gibco, Thermo Fisher Scientific, Waltham, MA, USA; DMEM/F-12 1:1 PAN-Biotech, Aidenbach, Germany). Cells were then cultured in serum-free medium (80 µl/cm²) for 24 h at 37 °C and 5% CO₂. Conditioned media (CM) were collected and centrifuged at 300 × *g* for 10 min to remove cells and subsequently at 14,000 × *g* for 10 min to remove debris. To reduce donor variability, CM from MDM derived from 8–12 healthy donors was pooled. CM was used for: (i) MESO culture and stimulation (morphology, MMT markers, functional assays), (ii) affinity-based proteomics analyses and (iii) validation experiments (ELISA, immunoblotting).

### Stimulation and culture of MESO

MESO were seeded at 5 × 10⁴ cells per well (24-well plates) in OCMI medium containing 5% FCS as described previously [17]. Cells were stimulated with CM from MDM / TAM conditions at a ratio of 1:3 for 72 h (functional assays) or 15 min (signaling analyses) at 37 °C, 5% CO_2_. Basal medium served as control (CM Ctrl). FCS was substituted to the serum-free CM to restore the final FCS concentration of 5% in the medium.

For inhibitor studies, MESO were pretreated for 1 h with SB431542 (10 µM; Sigma-Aldrich), BIRB796 (10 µM; Cayman Chemical, Ann Arbor, MI, USA), trametinib (30 nM; Cayman Chemical), or DMSO as solvent control prior to CM stimulation. For neutralization experiments, we used antibodies selectively binding to IL-1α, IL-1β or TNFα thereby blocking cytokine from interacting with its receptors and neutralizing downstream inflammatory signaling cascades. m1-MDM CM was preincubated for 1 h with 1 µg/ml mouse anti-human IL-1α antibody (Ab) (#MAB200-SP, Bio-Techne, Minneapolis, MN, USA), or mouse IgG (ms IgG) as isotype control (Jackson ImmunoResearch, Cambridgeshire, UK), 25 µg/ml anti-human IL-1β antibody canakinumab (#MAB10349-SP, R&D Systems, Bio-Techne, Minneapolis, MN, USA), 10 µg/ml anti-human-TNFα antibody infliximab (Remsima; Celltrion Healthcare, kind gift from Christian Bauer, Clinic for Gastroenterology, Endocrinology, Infectiology and Metabolism, Philipps University Marburg, Germany) before application. Recombinant human (rh) proteins were used at defined concentrations to assess individual factor effects as follows: 5 ng/ml IL-1α, 2.5 ng/ml IL-1β (both ImmunoTools, Friesoythe, Germany), 5 ng/ml TGFβ1, 10 ng/ml TNFα, 20 ng/ml LIF, 100 ng/ml MDK (all PeproTech), 100 ng/ml VEGF165 (Proteintech, Planegg-Martinsried, Germany), 100 ng/ml SEMA7A, 100 ng/ml IFN-y (Biomol, Hamburg, Germany), and 10 µM EDN1 (MedChemExpress LLC, Monmouth Junction, NJ, USA).

### Cell viability assay

Cell viability of MESO after 3 d exposure to m1-MDM CM combined with inhibitors (10 µM SB431542, 10 µM BIRB796, 30 nM trametinib) or solvent control (DMSO) was evaluated by staining with 3-[4,5-dimethylthiazol-2-yl]-2,5-diphenyl tetrazoliumbromid (MTT, Invitrogen, Carlsbad, CA) according to the manufacturer.

### Quantification of morphological changes

MESO morphology was quantified by measuring the reduction of cell circularity as an indicator for mesenchymal differentiation using ImageJ software (v1.54 f). Microscopic images were acquired 24 h after CM stimulation to allow reliable identification of cell borders using a Leica DMI3000B microscope (Leica, Wetzlar, Germany). Circularity was determined for 10 cells in three random fields per condition.

### Collagen contraction assays

After three days of CM treatment, MESO were harvested and embedded in collagen gels consisting of 1 µg/ml rat-tail collagen I (ibidi, Gräfelfing, Germany) in OCMI/5% FCS media. After O/N polymerization at 37 °C, 5% CO_2_, gels were detached from the well margin and overlayed with OCMI/5%FCS media. Gel shrinkage was analyzed using ImageJ software by measuring the gel area of each condition after 3 days, normalized to cell-free controls.

### Transwell macrophage-MESO coculture model

The impact of differently polarized macrophages on MESO morphology was additionally tested in a Transwell coculture system with a 0.4 µm pore size (Corning, Thermo Fisher Scientific, Waltham, MA, USA). Monocytes isolated from LRS chambers from healthy donors were plated in the bottom well of the Transwell system and differentiated into m1-, m2- and asc-MDM as described above. After 7 days of MDM differentiation, primary MESO from OC patients were added to the upper Transwell insert, and MDM-MESO coculture was performed in OCMI/5% FCS media for 3 days at 37 °C, 5% CO_2_. Changes in MESO morphology were analyzed by microscopic visualization using a Leica DMI3000B microscope and the Leica LAS X software platform (Leica, Wetzlar, Germany).

### Transmesothelial tumor invasion assay

Tumor cell invasion through a mesothelial layer was analyzed in a Transwell assay as described previously [17]. Briefly, MESO were plated on Transwell inserts (8 µm pores, Corning, Thermo Fisher Scientific) coated with 5 µg/cm^2^ rat-tail collagen I, and cultured with CM for 4 days at 37 °C, 5% CO_2_.

The primary OC cell line OCMI OC_58 was labeled with 10 µM CellTracker green (Invitrogen, Thermo Fisher Scientific, Waltham, MA, USA) and 5 × 10^4^ cells were added per inserts under serum-free conditions. OCMI containing 5% FCS was applied to the bottom well as a chemoattractant. For each condition, a negative control with serum-free OCMI as a chemoattractant was included. Tumor cells were allowed to invade through the mesothelial layer and the underlaying collagen matrix for 24 h at 37 °C and 5% CO_2_. Invaded cells were quantified microscopically in six visual fields per condition using a Leica DMI3000B microscope and ImageJ software. The respective tumor cell invasion in the negative control was subtracted from each condition.

### Immunofluorescence staining of ZO1 in MESO

The tight junction protein zonula occludens 1 (ZO1) was stained in MESO stimulated with CM of different conditions to assess junctional integrity. After three days of culture, MESO were fixated with 4% formaldehyde, permeabilized with 0.3% Triton X-100 and blocked in 1% BSA before immunofluorescent staining was performed with anti-ZO-1, Alexa Fluor 488–conjugated antibody (1:100 dilution, #339188, Invitrogen). Microscopic imaging was carried out on a Leica DMI3000B microscope and the Leica LAS X software platform.

### Immunohistochemistry

For immunohistochemistry staining of peritoneal tissue sections from OC patients, a heat-induced epitope retrieval with Citrat for IL-1α and with EDTA for CD68 was applied. Staining was done using a Dako Autostainer Link 48. After effectively blocking endogenous peroxidase activity, sections were incubated for 45 min with rabbit polyclonal IL-1α antibody (1:100, #16765-1-AP, Proteintech), and mouse monoclonal anti-CD68 antibody (1:100; #087601-2, Agilent, Santa Clara; CA). Following this step, sections were washed and treated with Dako REAL EnVision HRP Rabbit/Mouse polymer (Agilent), designed to react with DAB chromogen, in accordance with the manufacturer’s protocol for mouse tumor staining and ABC-method for human tissue.

### RTq-PCR

RNA isolation, cDNA synthesis and qPCR were performed as described previously [17] on a Quantabio Q 4 channel instrument (Quantabio, MA, USA). Expression levels were normalized to RPL27 and calculated using the 2⁻ΔΔCt method. Primer sequences are listed in Supplementary Table 3.

### Immunoblotting

Protein extracts were generated using RIPA lysis buffer (10 mM Tris-HCl, 150 mM NaCl, 1% NP40, 0.1% SDS, 1 mM EDTA, 1% Sodiumdeoxycholate) and analyzed by standard immunoblotting using the following antibodies: p-p44/42 MAPK (#4370, Cell Signaling Technology, Leiden, The Netherlands), p44/42 MAPK (#9107, Cell Signaling Technology), p-p38 MAPK (#4511, Cell Signaling Technology), p38 MAPK (#9228, Cell Signaling Technology), p-SMAD2 (#3108, Cell Signaling Technology), SMAD2 A11 (#sc-393312, Santa Cruz Biotechnology, Dallas, TA, USA), TGF-β RI/ALK-5 (#MAB5871, Bio-Techne, Minneapolis, MN, USA), GAPDH (#G9545, Sigma-Aldrich, Merck, Darmstadt, Germany), α-rabbit IgG HRP-linked polyclonal antibody (#7074, Cell Signaling Technology), α-mouse IgG HRP-linked polyclonal antibody (#7076, Cell Signaling Technology) and rat IgG Horseradish Peroxidase-conjugated Antibody (#HAF005, R&D Systems, Bio-Techne).

### ELISA

Cytokine concentrations (IL-1α, IL-1β, TGFβ1, TNFα, M-CSF and GM-CSF) in CM of macrophages and MESO were quantified using ELISA kits (all R&D Systems, Bio-Techne, Minneapolis, MN, USA) according to manufacturer instructions.

### Bulk RNA-seq of MESO

RNA from MESO (*n* = 3 OC patients) treated with m1-MDM CM or CM Ctrl for 72 h was processed using the Lexogen Quantseq FWD mRNA-seq v2 Library Prep Kit with UMI (Lexogen, Vienna, Austria) according to manufacturer’s instructions and sequenced on an Illumina NextSeq 550 platform at the Genomics Core Facility at the Medical Department of Marburg University. Data processing was performed as described previously [57]. Briefly, raw reads were aligned using STAR 2.7.10a [58] against the human genome and gene assembly from Ensembl version 108 [59]. Reads were deduplicated based on alignment position and unique molecular read identifier using UMI-tools [60]. Gene expression was quantified using custom python scripts. Only protein-coding genes were considered for further analysis. Differential gene expression was tested using edgeR [61](version 4.2.0)’s glmTreat modeling, blocked on donor identity, for true |log2FC | > 1.0 at an FDR < 0.05. Data are available at ArrayExpress (E-MTAB-16779).

### scRNA-seq of TAM subsets

scRNA-seq was performed with matched pairs of cryopreserved omTAM and ascTAM samples isolated from the metastatic omentum tissue and the ascites of three high-grade OC patients (for patient characteristics see Supplementary Table S2). Purity was >95% for CD14+ cells. Only TAMs with <5% aggregates and >70% vitality were processed with Single Cell Parse Cell Fixation Kit (Parse Biosciences, Seattle, WA, USA) according to the instructions of the manufacturer. scRNA-seq was generated using the Evercode™ WT Mini v2 kit (Parse Biosciences) and analyzed on an Illumina NovaSeq 6000 by the Genomics Core Facility at the Medical Department of Marburg University. Raw reads were aligned and quantified using the STARSolo mode of STAR (2.7.11b) and further analyzed using scanpy 1.10.2. Genes were filtered for a minimum of 30 cells and a minimum of 100 reads. Cells were filtered to a minimum of 1000 reads. Non macrophage cells were identified by UMAP visualization and manual identification based on T cell and mesothelial markers. PCA was corrected on sample identity (donor & cell source) using harmony_py (version 0.0.10) [62]. Clustering was performed using the Louvain algorithm [63]. Gene expression matrices and cell type annotation have been deposited at EBI ArrayExpress (accession number E-MTAB-16778).

M1 and M2 gene scores were based on predefined gene sets derived from previously published bulk RNA-seq datasets of our group [34], and the MMT driver score was calculated based on a ChatGPT-generated list of EMT-associated macrophage genes (Supplementary Table S1), which was verified using the corresponding entries in the GeneCards database (www.genecards.org). External MESO scRNA-seq data from OC patients (E-MTAB-13498) [35] were reanalyzed for validation of m1-MDM-induced mesenchymal gene signatures (Supplementary Table S1) obtained from the bulk RNA-seq experiment described above. In addition, the expression of IL1R1-related genes (Supplementary Table S1) differential in the bulk RNA-seq of m1-MDM-treated MESO was visualized in the scRNA-seq MESO data.

### Affinity proteomics of macrophage secretomes

CM of m1-MDM and asc-MDM (matched pairs, *n *= 4 healthy donors) were analyzed using the Olink Explore 3072 platform at the Core Facility Translational Proteomics at the Medical Department of Marburg University, following the Olink standard protocol (v1.5, 2022-12.21). Olink Explore uses Proximity Extension Assays (PEA) technology [64] which has been optimized for high-throughput analysis by Next-Generation Sequencing [65]. Samples were randomized as members of a larger set of conditioned media analyzes on 96-well plates and processed in one batch. Generated libraries were sequenced at the Genomics Core Facility of the Department of Medicine at Marburg University. Protein expression was reported as Normalized Protein eXpression (NPX) values (arbitrary units in log₂ scale). For proteins present in more than one Olink Explore panel, median NPX values were used for bioinformatic analyses (Supplementary Table S4, complete data set). M1-MDM-specific proteins were identified by difference NPX values (∆NPX) for samples compared to asc-MDM CM. Fold change (FC) values (2∆NPX) were calculated as the median of *n* = 4 biological replicates. False discovery rates (FDR) were assessed using the Benjamini-Hochberg method on nominal *p* values determined by unpaired *t* test of FC values. EMT-related secreted proteins were identified via database filtering using the search term ‘secreted’ and ‘EMT-related’ in the genecards.org database (Supplementary Table S5).

### Label-free mass spectrometry based proteomic analysis of omTAM and ascTAM secretomes

For orthogonal validation, label-free mass spectrometry (MS) analyses of paired omTAM and ascTAM CM from six OC patients were performed. Sham media only briefly exposed to omTAM and ascTAM, respectively, was processed in parallel, pooled from 6 patients and used as background (*n* = 2). Proteins present in a volume of 100 μl of CM were digested as reported [66]. Concentration of digested peptides was quantified using the fluorometric Pierce Quantitative Peptide Assay, and sample volumes were adjusted to ensure equal concentration across all samples. Purified peptides were analyzed by liquid chromatography–tandem MS carried out on a Bruker Daltonics timsTOF Ultra connected to a Bruker Daltonics nanoElute instrument. Approximately 50 ng of peptides were loaded onto a C18 precolumn (Thermo Trap Cartridge 5 mm, μ-Precolumn™ Cartridge/PepMap™ C18, Thermo Scientific) and eluted in backflush mode with a gradient from 98% solvent A (0.15% FA) and 2% solvent B (99.85% ACN and 0.15% FA) to 10% solvent B over 5 min, continued from 10% to 35% solvent B over 25 min, then from 35% to 95% solvent B over another 1 min on a reverse-phase high-performance liquid chromatography (HPLC) separation column ((PepSep Ultra C18, 1.5 µm, 75 µm × 25 cm; Bruker Daltonics) with a flow rate of 300 nl/min. The analytical column outlet was connected to the mass spectrometer via a 20 µm CaptiveSpray emitter. Data were acquired in data-independent acquisition (DIA) mode using the manufacturer’s default method. MS1 spectra were recorded at a resolution of 45,000 across an m/z range of 100–1700. Ion mobility separation was performed over a 1/K₀ range of 0.64–1.45 V·s/cm² with a ramp time of 100 ms. DIA scans were acquired using 24 fixed isolation windows (25 m/z each), covering a precursor range of 400–1000 m/z. Peptide spectrum matching and label-free quantitation were subsequently performed using DIA-NN 2.5.1 Academia (Data-Independent Acquisition by Neural Networks) [67] in a library-free search against the Human Uniprot.org database (20,405 reviewed Swiss-Prot entries; January 2026). In brief, output was filtered to a 1% false discovery rate (FDR) at the precursor level. Deep learning was used to generate an in silico spectral library for the library-free search. Fragment m/z was set to a minimum of 200 and a maximum of 1800. In silico peptide generation allowed for N-terminal methionine excision, tryptic cleavage following K*, R*, a maximum of one missed cleavage, as well as a peptide length requirement of seven amino acids minimum and a maximum of 30. Cysteine carbamidomethylation was included as a fixed modification and methionine oxidation (maximum of two) as a variable modification. Precursor masses from 300 to 1800 m/z and charge states up to four were considered. Downstream data processing and statistical analysis were carried out using the Autonomics package developed in-house (Version autonomics_1.21.2) [68]. Briefly, peptide/spectrum-matching using DIA-NN was filtered to a 1% FDR at the precursor level and only proteins groups with a q value < 0.01 were retained for further analysis. Differential abundance of protein groups between omTAM and ascTAM CM was evaluated by Autonomics employing a Bayesian moderated *t*-test as implemented by limma [69]. Differentially abundant proteins were filtered by fold-change (FC) > 1 and FDR cut-off < 0.05. Additionally, only secretome proteins according to Human Protein Atlas database (2,520 entries; Version 25.1) [70] and EMT-related proteins (GeneCards, 1504 entries under “EMT-related, protein coding, extracellular space”; Version 6.1, LifeMap Sciences) were selected for further analysis. Protein list, abundance changes and statistical evaluation are depicted in Supplementary Table S6. Raw MS data, processing and statistical analysis have been deposited at ProteomeXchange Consortium via the MassIVE partner repositories (ProteomeXchange: PXD080856; MassIVE ID: MSV000102418. http://proteomecentral.proteomexchange.org).

### Statistical analysis

Statistical analyses were performed using GraphPad Prism v10.2.0. Data are presented as mean ± SD. Statistical tests included one-sample *t*-tests, paired *t*-tests, unpaired *t*-test and one-way ANOVA with Tukey’s post hoc test, as appropriate and indicated in the figure legends. Significance thresholds were: *p < 0.05, **p < 0.01, ***p < 0.001, ****p < 0.0001. Gene ontology analysis was performed using the Gene Ontology resource [71] at http://geneontology.org.

## Supplementary information

Supplementary Figures

Supplementary Tables

Original Western Blots

## Data Availability

Bulk RNA-seq data were deposited at EBI ArrayExpress (accession number E-MTAB-16779). For scRNA-seq, gene expression matrices and cell type annotation have been deposited at EBI ArrayExpress (accession number E-MTAB-16778). Other data supporting the findings are available in the main article and supplementary files. For label-free MS data, the full list of DIA-NN settings are depicted in the “report.log.txt”; the code for data processing and statistical analysis are found in “mdmev-003-statistics.zip”, along with the mass spectrometric raw data (““mdmev-003-rawfiles.zip”) and UniProt database, all accessible at the ProteomeXchange Consortium with dataset identifier: PXD080856, via the MassIVE partner repository (https://massive.ucsd.edu/, MassIVE ID: MSV000102418; 10.25345/C5BC3TB5C).
