# Supplementary material for: A pro-inflammatory metastasis-associated macrophage subset induces tumor-promoting mesothelial cell conversion in ovarian cancer via IL-1α secretion
Source: Cell Death Dis. 2026 Sep 12;17(1):794. doi: 10.1038/s41419-026-09280-1 (PMC13570891; doi:10.1038/s41419-026-09280-1)
Supplement: Supplementary file 1 [file 41419_2026_9280_MOESM1_ESM.docx]

**Supplementary Information**

**A pro-inflammatory metastasis-associated macrophage subset induces tumor-promoting mesothelial cell conversion in ovarian cancer via IL-1α secretion**

**Authors**

Sophie Heidemann^1^, Florian Finkernagel^2^, Anna Mary Steitz^1^, Julie Koennecke^1§^, Franziska Werner^1$^, Vanessa M. Beutgen^3^, Witold Szymanski^3^, Johannes Graumann^3^, Julia Teply-Szymanski^4^, Andrea Nist^2^, Thorsten Stiewe^2,5^, Corinna U. Keber^4^, Niklas Gremke^6^, Uwe Wagner^6^, Carsten Denkert^4^, María Gómez-Serrano^7^, Elke Pogge von Strandmann^7^, Rolf Müller^1^, Silke Reinartz^1^*

^1^ Clinic for Gynecology, Gynecological Oncology and Gynecological Endocrinology, Center for Tumor Biology and Immunology, Philipps-Universität Marburg, 35043 Marburg, Germany

^2^Genomics Core Facility, Philipps-Universität Marburg, 35043 Marburg, Germany

^3^Institute of Translational Proteomics & Core Facility Translational Proteomics, Philipps-Universität Marburg, 35043 Marburg, Germany

^4^Institute for Pathology, Philipps-Universität Marburg and University Hospital Marburg (UKGM), 35043 Marburg, Germany

^5^Institute of Molecular Oncology, Member of the German Center for Lung Research (DZL), Philipps-Universität Marburg, 35043 Marburg, Germany

^6^Clinic for Gynecology, Gynecological Oncology and Gynecological Endocrinology, University Hospital Marburg (UKGM), 35043 Marburg, Germany

^7^Institute for Tumor Immunology, Center for Tumor Biology and Immunology, Philipps-Universität Marburg, 35043 Marburg, Germany

^§^*Current address*: Institute of Pharmaceutical Biology, Goethe University Frankfurt, 60438 Frankfurt am Main, Germany

^$^*Current address*: Clinical Cooperation Unit Applied Tumor Immunity, German Cancer Research Center (DKFZ), Heidelberg, Germany

***Correspondence: [silke.reinartz@uni-marburg.de](mailto:silke.reinartz@uni-marburg.de)**

**Supplementary Figures**

**Figure S1. Density estimates of M2, M1 and MMT driver score per Louvain cluster of omTAM and ascTAM.** **(A)** Heatmap illustrating the Top 5 marker genes in each Louvain cluster. Calculation was performed via the scanpy.rank_genes_groups (method='t-test') method. Score was normalized to 0…100 for each gene. **(B-D)** Histograms displaying density estimate of M2 **(B)**, M1 **(C)** and MMT driver score **(D)** stratified by Louvain cluster. Scores are defined by expression of selected genes for M1, M2 and MMT drivers (Supplementary Table S3). Scores are calculated as sum of (normalized, log2 transformed) genes. *(Right, lower panel)* UMAP projection of Louvain clustering of scRNA Seq data of om/ascTAM from OC patients (n=11 cluster, as in Fig. 1). Cluster names indicate the main cellular source in the cluster (omTAM and ascTAM). Clusters labeled with om/ascTAM comprise omTAM and ascTAM with similar marker gene expression.

**Figure S2.** **Analysis of morphologic changes of MESO after coculture with m1-, m2- and asc-MDM in a Transwell system. (A)** After differentiation of m1-, m2- and asc-MDM (n=3 donors) for 7 days in the bottom well of a Transwell coculture plate, patient-derived MESO were added to the upper Transwell insert (0.4 µm pore size) and cocultured with MDM for 3d in OCMI/5% FCS medium. Microscopic images of MESO morphology (upper insert) are depicted for each coculture condition and MDM donor. MESO cultured in the absence of MDM (MESO only) served as control. (Scale bar = 15 µm). **(B)** Exemplary microscopic images of m1-, m2- and asc-MDM (bottom well) of one donor are presented before (0 d) and after 3 d of MESO coculture. (Scale bar = 20 µm).

**Figure S3.** **Validation of** **ERK/p38 MAPK and TGFβ/SMAD pathway activation in MESO. (A)** Detection of p38 and ERK activation in MESO exposed to CM of asc-MDM, m1-MDM, patients’ TAM (om/ascTAM, patients OC_347 and 349) and Ctrl for 15 min. Alternative immunoblot pictures are presented from a different experiment in addition to Fig. 5A. **(B)** Activation of p38 in MESO by stimulation with m1-MDM CM for 15min was blocked by preincubation with 10 µM BIRB796 (n=3). **(C)** Activation of ERK in MESO by stimulation with m1-MDM CM for 15min was blocked by preincubation with 30 nM trametinib (n=3). **(D)** Activation of SMAD2 in MESO by stimulation with m1-MDM CM for 15min was blocked by preincubation with 10 µM SB431542 (n=2). DMSO serves as solvent control. Stimulation with asc-MDM CM and CM Ctrl were included as controls. **(E, F)** Preincubation of MESO with TGFβ inhibitor SB431542 did not affect m1-MDM CM induced p38 activation (n=3) **(E)** and ERK activation (n=4) **(F)**. **(G)** Impact on ERK/p38 MAPK and TGFβ/SMAD pathway activation on the expression of mesenchymal marker FN1 in MESO. MESO were pretreated with inhibitors trametinib (n=3), BIRB769 (n=4), SB431542 (n=4) or solvent control (DMSO) before m1-MDM CM were added to the cultures for 72h. FN1 expression was determined by RTq-PCR. Dotted line separates data from different inhibitor conditions. **(H)** MTT-based cell viability assay of MESO treated with m1-MDM CM and inhibitors (10µM BIRB796 n=6; 30 nM trametinib n=5; 10µM SB431542 n=3) or solvent control (DMSO) for 3 days. Absorption of MTT (A560-A690) is calculated relative to MESO treated with m1-MDM +DMSO showing no significant influence of inhibitors on cell viability. **(I)** Expression of the TGFβ2 isoform was measured in MESO treated for 72h with CM of matched pairs of ascTAM and omTAM (n=4) by RT-qPCR. MESO stimulated with CM Ctrl serves as control. Plots show the mean ± SD. One-sample t-tests was performed in Fig. S3 B-F, H. Comparison of conditions was performed by two-sided, paired t test for Fig. S3 G,I). *p<0.05, **p<0.01, ***p<0.001, ****p<0.0001.

**Figure S4.** **Impact of macrophage-derived factors identified by affinity proteomics on MESO phenotype. (A-D)** Representative microscopic images of MESO after 72h-exposure to recombinant, m1-MDM-specific factors identified by PEA-based affinity proteomics. **(A)** rh IL-1α, IL-1β (alone and in combination), a mixture of rh LIF, MDK, SEMA7A, and solvent control diluted in CM Ctrl, **(B)** rh TNFα, **(C)** rh IFNγ, **(D)** rh EDN1 and **(E)** rh VEGF165. Scale bar = 20 µm. **(F)** Quantification of collagen contraction by MESO treated with recombinant factors (alone and as mixtures of multiple factors) for 72h as indicated, including solvent control treated MESO. Bars show the mean ± SD.

**Figure S5.** **Comparative analysis of MESO alterations induced by macrophage-derived IL-1α,** **IL-1β and TNFα. (A)** Representative microscopic images of MESO after 72h-exposure to m1-MDM CM which was pretreated with 25 µg/ml neutralizing IL-1β antibody canakinumab or solvent control. Scale bar = 20 µm. **(B)** Impact of IL-1β blocking antibody canakinumab on mesenchymal transition induced by m1-MDM CM in MESO. Mesenchymal (*Left: FN1*) and epithelial (*Left*: *CDH1*) marker gene expression was analyzed by RTq-PCR in MESO treated for 72h with CM from m1-MDM with canakinumab or solvent control. **(C)** Representative microscopic images of MESO after 72h-exposure to m1-MDM CM which was pretreated with 10 µg/ml neutralizing TNFα antibody infliximab or solvent control. Scale bar = 20 µm. **(D)** Representative picture of a collagen contraction assays performed with MESO treated with CM from m1-MDM with canakinumab (*left panel)* or infliximab (*right panel)* for 72h. **(E-G)** Impact of macrophage-derived IL-1α on ERK **(E)**, p38 **(F)** and TGFβ/SMAD activation **(G)**. MESO were stimulated for 15min with either recombinant IL-1α and solvent control or m1-MDM CM blocked with neutralizing IL-1α antibody and ms IgG as control. **(H)** IL-1β concentrations in CM of asc-MDM compared to m1-MDM (*Left:* n=3 donors) and in CM of paired ascTAM / omTAM (*Right:* n=5 patients) as determined by ELISA. **(I)** TNFα concentrations in CM of asc-MDM compared to m1-MDM (n=6 donors) as determined by ELISA. Plots show the mean ± SD. Comparison of conditions was performed by one-sample t-test (Fig. S5 E,F,I) and two-sided, paired t test (Fig. S5 E,F,H). *p<0.05, **p<0.01, ***p<0.001, ****p<0.0001; ns = non-significant; n.d. = not-detectable.

**Figure S6.** **IL-1α-mediated regulation of** **TGFβ1/2 expression in MESO. (A)** TGFβ1 concentrations in CM of MESO treated with recombinant IL-1α or PBS as solvent control for 72h (n=5). **(B-C)** RNA expression of *TGFB1* **(B)** and *TGFB2* **(C)** in MESO after 72h-exposure to m1-MDM CM pretreated with neutralizing IL-1α antibody or ms IgG as control. ascMDM CM is included as control. **(D)** Mesenchymal marker gene expression (*FN1*) was analyzed by RTq-PCR in MESO treated for 72h with either recombinant cytokines IL-1α and TGFβ1 (alone or combined; *Left*) or m1-MDM CM blocked with neutralizing IL-1α antibody (ms IgG as control) and TGFβ inhibitor SB413162 (*Right*). Plots show the mean ± SD. Comparison of conditions was performed by two-sided, paired t test (Fig. S6 A). Multiple-group-comparison were analyzed with ANOVA (one-way) and Tukey's post hoc test (Fig. S6 B-D). *p<0.05, **p<0.01, ***p<0.001, ****p<0.0001. ns = non-significant.

**Figure S7. Generation of m1-, m2- and asc-MDM from healthy donors. (A)** GM-CSF and M-CSF concentrations in ascites of high-grade OC patients were determined by ELISA (n=38 patients). Comparison was performed by Wilcoxon test of paired samples. ****p<0.0001. **(B)** Representative images of m1-, m2-, and asc-MDM after differentiation for 7d. Scale bar = 10µm. **(C)** Phenotypic characterization of m1-, m2, and asc-MDM after 7d by flow cytometric analysis of surface markers CD14, CD86, CD163, HLA-DR and CD16. Expression was calculated as percentage of positive cells (for CD14, CD163 and CD16) and mean fluorescence intensity (MFI) for n=11 donors (m1- and asc-MDM) and n=3 donors (m2-MDM). Plots show the mean ± SD. Comparison was performed by Wilcoxon test of paired samples for Fig. S7 A. Multiple-group-comparison were analyzed with ANOVA (one-way) and Tukey's post hoc test (Fig. S7 C). **p<0.01, ***p<0.001, ****p<0.0001.
